# Prediction of acute appendicitis among patients with undifferentiated abdominal pain at emergency department

**DOI:** 10.1186/s12874-021-01490-9

**Published:** 2022-01-14

**Authors:** Dai Su, Qinmengge Li, Tao Zhang, Philip Veliz, Yingchun Chen, Kevin He, Prashant Mahajan, Xingyu Zhang

**Affiliations:** 1 Department of Health Management and Policy, School of Public Health, Capital Medical University, Beijing, China; 2 Department of Systems, Populations, and Leadership, University of Michigan School of Nursing, Ann Arbor, USA; 3 Department of Biostatistics, University of Michigan School of Public Health, Ann Arbor, USA; 4 Department of Epidemiology and Biostatistics, West China School of Public Health School, Sichuan University, Chengdu, China; 5 Department of Health Management, School of Medicine and Health Management, Tongji Medical College, Huazhong University of Science and Technology, Wuhan, China; 6 Research Center for Rural Health Services, Hubei Province Key Research Institute of Humanities and Social Sciences, Wuhan, China; 7 Department of Emergency Medicine, University of Michigan School of Medicine, Ann Arbor, USA; 8 Thomas E. Starzl Transplantation Institute, University of Pittsburgh Medical Center, Pittsburgh, USA

**Keywords:** Acute appendicitis, Emergency department, Machine learning, Prediction modelling, Precision health

## Abstract

**Background:**

Early screening and accurately identifying Acute Appendicitis (AA) among patients with undifferentiated symptoms associated with appendicitis during their emergency visit will improve patient safety and health care quality. The aim of the study was to compare models that predict AA among patients with undifferentiated symptoms at emergency visits using both structured data and free-text data from a national survey.

**Methods:**

We performed a secondary data analysis on the 2005-2017 United States National Hospital Ambulatory Medical Care Survey (NHAMCS) data to estimate the association between emergency department (ED) patients with the diagnosis of AA, and the demographic and clinical factors present at ED visits during a patient’s ED stay. We used binary logistic regression (LR) and random forest (RF) models incorporating natural language processing (NLP) to predict AA diagnosis among patients with undifferentiated symptoms.

**Results:**

Among the 40,441 ED patients with assigned International Classification of Diseases (ICD) codes of AA and appendicitis-related symptoms between 2005 and 2017, 655 adults (2.3%) and 256 children (2.2%) had AA. For the LR model identifying AA diagnosis among adult ED patients, the c-statistic was 0.72 (95% CI: 0.69–0.75) for structured variables only, 0.72 (95% CI: 0.69–0.75) for unstructured variables only, and 0.78 (95% CI: 0.76–0.80) when including both structured and unstructured variables. For the LR model identifying AA diagnosis among pediatric ED patients, the c-statistic was 0.84 (95% CI: 0.79–0.89) for including structured variables only, 0.78 (95% CI: 0.72–0.84) for unstructured variables, and 0.87 (95% CI: 0.83–0.91) when including both structured and unstructured variables. The RF method showed similar c-statistic to the corresponding LR model.

**Conclusions:**

We developed predictive models that can predict the AA diagnosis for adult and pediatric ED patients, and the predictive accuracy was improved with the inclusion of NLP elements and approaches.

**Supplementary Information:**

The online version contains supplementary material available at 10.1186/s12874-021-01490-9.

## Background

AA is one of the most common surgical emergencies but has a high rate of misdiagnosis in the United States [1]. It is also the second most common condition among pediatric malpractice claims and third for adult malpractice claims [2, 3]. The lifetime risk of developing appendicitis is approximately 7% and usually requires surgical treatment [4, 5]. The annual national rate of AA is up to 13/100,000 patients [6], but the diagnosis of AA is missed at a rate of 3.8-15% for children and 5.9-23.5% for adults during ED visits [7–11]. While the clinical diagnosis may be straightforward in patients who present with classic signs and symptoms, atypical presentations may result in diagnostic confusion and delay in treatment. The diagnosis of AA can be challenging even in the most experienced hands. Abdominal pain is the primary presenting complaint of patients with AA. Accurately identifying AA among patients with undifferentiated symptoms at emergency visits can potentially improve the patient safety and health care quality.

Technological innovations that employ NLP and machine learning (ML) techniques can be used to extract useful features from the complex structured and unstructured retrospective electronic health records (EHRs) data to potentially replicate the clinician’s thought process at ED presentation. These features can be used to accurately identify a patient’s diagnosis, which has the potential to improve ED patient safety [12]. Among ED patients, the ML and NLP techniques have proven useful in better understanding the associated factors related to ED health outcomes, such as hospitalization and medical resource utilization, and thus, they can be used to improve predictive performance for these outcomes [13–16]. However, few studies have focused on using NLP and ML to identify a patient’s diagnosis and potential misdiagnosis [17].

The aim of the study was to develop ML and NLP models as an assistive technique to predict AA among patients with undifferentiated symptoms at ED visits. We hypothesize that the prediction accuracy can be improved with the inclusion of NLP elements.

## Methods

### Study design and setting

We carried out the study on combined data from the ED component of the NHAMCS datasets (2005-2017). The Centers for Disease Control and Prevention (CDC) has been publishing the NHAMCS data annually since 1992, which collects data on the utilization and provision of ambulatory care services in hospital emergency and outpatient departments. The ED component of NHAMCS is a multistage, stratified probability sample of ED visits from 300 hospital-based EDs each year, which was randomly selected from about 1900 geographically defined areas across the United States, administered by the National Center for Health Statistics (NCHS) [18]. The NHAMCS is a public use dataset that does not require ethical committee or institutional review board approval.

### Definition of appendicitis

AA in this study was defined by the ICD, 9^th^ and 10^th^ Revision, Clinical Modification (ICD-9-CM and ICD-10-CM) diagnosis codes from category 540-542 (ICD-9-CM) and K35-K37 (ICD-10-CM), which refers specifically to essential (or primary) appendicitis [19]. Along with the implementation of ICD-10-CM since 2015, an ICD-10-CM category of K35-K37 was used to define the diagnosis of primary appendicitis, which is equivalent to the ICD-9-CM category 540-542, according to the ICD-10-CM General Equivalence Mapping (GEM), a crosswalk between the two code standards maintained by the Centers for Medicare and Medicaid Services (CMS) and the CDC.

### Study patients

A total of 356,333 patient visits were included in the ED component of the survey datasets from 2005 to 2017. According to the ICD-9-CM and ICD-10-CM, we selected 40,041 patients from which were assigned a ICD code of AA and showed at least one symptoms (abdominal pain, constipation, diarrhea, fever, and nausea and/or vomiting) associated with appendicitis during the ED (Tables S1 and S2). We then divided the patients into two groups by age (>=18 years old or <18 years old), respectively: the adult group (*N* = 28657, 71.57%) and the pediatric group (*N* = 11384, 28.43%).

### Study variables

#### Outcomes

The primary outcome variable for this study was whether the eventual diagnosis was AA during an ED visit. The outcome variable was assigned a value of 1 if the eventual diagnosis was appendicitis, while symptoms associated with appendicitis but not assigned an ICD code of AA was assigned a value of 0.

#### Predictors

The predictors for ML models were chosen from routinely available data at ED components using a priori knowledge [20–22]. This study classified predictors into two categories, structured variables and unstructured variables.

Specifically, the structured predictors included: sex, race, ethnicity, type of residence, insurance, visit year, month and day, arrival time, initial vital signs (body temperature, respiratory rate, systolic and diastolic blood pressure, pulse oximetry), 5 point triage level (immediate, emergent, urgent, semi-urgent, nonurgent), pain scale (mild, moderate, very severe), 72 hour revisit, whether the visit was related to an injury, poisoning, or adverse effect of medical treatment, whether is injury/poisoning intentional, and the diagnostic services (any laboratory tests or imaging tests) provided.

Unstructured data included up to three reasons for visiting the ED and three causes of injury recorded by the providers for each patient in the triage notes; the limit of three was by design of the NHAMCS. The reason for visit classification system derived by the NCHS is a modular framework into which the reason for visit is broadly categorized as a type of complaint (e.g., symptoms, diseases, injury) and a methodology for systematically recording these complaints within a specific organ or area of the body. The system then records the complaint in a pre-specified fashion according to an alphabetical index of complaints (for example, “eye pain” is changed to “pain, eye”) while maintaining the emphasis on the patient’s lay terminology rather than a clinician’s translation of the patient’s reason for the visit.

#### Missing values

Before statistical modelling, the k-nearest neighbors (k-NN) approach was used to impute missing data for most predictors. For a given patient with missing values, the k-NN method identified the k-nearest patients based on Euclidean distance. Using these patients, missing values were then replaced using a majority vote for discrete variables and weighted means for continuous features. One advantage of using this method is that missing values in all features are imputed simultaneously without the need to treat features individually [23].

### Statistical analysis

#### NLP

NLP is a field of Artificial Intelligence (AI) that gives the machines the ability to read, understand and derive meaning from human languages; in NLP there are many techniques to vectorize human languages -- either a word, a sentence, a paragraph, or even a document [24]. Since the unstructured variables in this study were all sentence forms, we carried out Doc2Vec method in Python, an embedded encoding method, for vectorization.

We first pre-processed the unstructured data, including word segmentation and removal of stop words. Then we used TaggedDocument in the gensim package to wrap the input sentence and change it to the input sample format required by Doc2Vec [25, 26]. After that, we loaded the Doc2vec model with window size of 3 and started training, and finally we mapped the unstructured data into 128-dimensional paragraph vectors and made further predictions.

The ML methods are data-driven and therefore rely on accurate data. Although there may be some misclassification in the survey data, in the 10% quality control sample of NHAMCS, the coding error rate was less than 1% [27]. Therefore, we established two main types of ML models to compare the predictive accuracy of being diagnosed of AA or not in a population of ED patients at the time of triage, using standard binary LR and RF methods in Python.

#### LR

LR is a member of the general linear model (GLM) family. It has the underlying assumption that the output follows a Bernoulli distribution with parameter p, where p is the probability of success (in our case the probability of appendicitis). This assumption is consistent with our appendicitis 0, 1 outcome. LR also uses a canonical link function in the form of: $\mathit{\log}\ \left(\frac{p_i}{1-{p}_i}\right)={e}^{x_i\beta }$. With a transformation we get ${p}_i=\frac{1}{1+{e}^{-{x}_i\beta }}$. Since the expectation of a Bernoulli distribution is p, the output of our predicted outcome is *p*_*i*_ for patient i.

The fitting of parameter *β* is done by a Maximum Likelihood Estimation (MLE); once the estimated betas are fitted, the predicted values can be calculated using the equation, ${p}_i=\frac{1}{1+{e}^{-{x}_i\beta }}$. In this study, the model building strategy for LR is direct (i.e., full, standard, or simultaneous), all predictors are entered into the equation at the same time.

In this study, we separately fitted three LR models for adults and children to determine the model’s predictive performance in identifying the eventual diagnosis: (1) models with structured variables only; (2) models with unstructured data; and (3) models with both structured and unstructured variables.

#### RF

We then employed a RF classifier, which has been widely used for classification and prediction in the fields of medicine and bioinformatics, to build prediction models of appendicitis in adults and children during ED visits [28–30]. The RF classifier is an ensemble of decision trees, and each tree learns from a randomly selected set of the training data. The information content of the decision tree classifier is derived from each attribute in the dataset. Therefore, the decision tree classification algorithm first selects the attribute with the most abundant information for classification. Sample training data sets are selected randomly and returned to ensure that the total size of each random sample is the same. For prediction, each decision tree is applied to the test set and the error is evaluated, and the final classification decision is made by majority voting on all decision trees.

Because of this non-parametric model setting, RF can be used in non-linear separable problems. However, this property is also problematic given that it makes the model very sensitive to noise. Therefore, before we carried out the classification, we did the data cleaning on the unstructured data. Firstly, Principal Component Analysis (PCA) was used to convert the original features to orthogonal ones. Then, based on the p-value of the Welch’s approximated t-test, we chose those features with statistical significance at a level of *p*<0.01, selecting 24 principal components out of the original 128 features. Based on the 20 structured and 24 unstructured datasets, we applied the standard RF classification package in Scikit-learn (Sklearn) on three models, the same as in LR, using 1000 trees in the RF implementation [31, 32]. The number of jobs to run in parallel was 90. The number of features selected at random at each tree node was set to log2*(n), where n was the total number of features [33].

#### Model evaluation

For both LR and RF models, we used 5-fold cross-validation to evaluate our model performance. Patients were randomly divided into 5 sets, and 4 of the 5 sets were used to train the models while the remaining set was used as the testing set. In the testing set, we measured the prediction performance of each model by computing (1) C-statistic (the area under the receiver operating curve, AUC) and (2) prospective prediction results (sensitivity, specificity, threshold, and accuracy). To address the class imbalance in the outcome, we chose the threshold of prospective prediction results based on the Receiver Operating Characteristics (ROC) curve (the value with the shortest distance to the perfect model) [15]. The C statistic informs in a single numerical value about the overall diagnostic accuracy of the index test. The C statistic ranges from 0.50 to 1.00, with higher values indicating better predictive models. Values above 0.80 indicate very good models, between 0.70 and 0.80 good models, and between 0.50 and 0.70 weak models. The average ROC curve was derived by comparing the prediction values from all 5 cross-validated testing sets. The ROC curve mentioned above is a curve that shows the overall performance of a specific model. Accordingly, with threshold from 0 to 1, we calculate the corresponding False Positive Rate (FPR) ($\frac{TP}{TP+ FN}$) and the True Positive Rate (TPR) ($\frac{FP}{FP+ TN}$). We then draw the point in a rectangular coordinator with the FPR as the horizontal coordinate and the TPR as the longitudinal coordinate. The better tendency the curves have to access the up-left corner of the coordinate, the better performance of the model. The perfect model should have a ROC curve as a line linking (0,0), (0,1), (1). The meaning of AUC is the possibility that while randomly choosing one positive patient and one negative patient, the score of the positive patient will be greater than the negative patient. So, the bigger the value, the better we have classified the two classes of patients.

##### Sensitivity

The recall depicts the ability of the model to search for all positive data. The calculation function is $R=\frac{TP}{TP+ FN}$.

##### Specificity

The precision depicts the ability of the model to search for all negative data. The calculation function is $P=\frac{TN}{TN+ FP}$.

## Results

Among the 40,441 ED patients with appendicitis-related symptoms between 2005 and 2017, 655 of 28,657 adults (2.3%) and 256 of 11384 pediatric patients (2.2%) had appendicitis (Table 1). Male appendicitis patients (3.5% for adults and 3.1% for pediatric patients) present at a higher proportion than female patients (1.7% for adults and 1.5% for pediatric patients). The proportion of appendicitis patients was highest among Asian adults (4.4%) and highest among white pediatric patients (2.7%). The highest proportion of triage level in adults and pediatric appendicitis patients was immediate (5.6 and 10.0%). The highest proportion of the pain level in the adults and pediatric patients with appendicitis was very severe (2.7 and 5.7%). A total of 2.4% of adult patients and 3.2% of pediatric patients who were provided diagnostic services were diagnosed as AA, which is higher than those adults patients (1.3%) and pediatric (0.5%) patients who did not have diagnostic services.

**Table 1:** Baseline characteristics of the United States appendicitis patients presenting to the ED NHAMCS 2005–2017

		All Adult N(%)	Adult Appendicitis N(%)	Adult Non-appendicitis N(%)	***p-value***^***1***^	All Pediatric N(%)	Pediatric Appendicitis N(%)	Pediatric Non-appendicitis N(%)	***p-value***^***1***^
		28657(100.0)	655(2.3)	28002(97.7)		11384(100.0)	256(2.2)	11128(97.8)	
Sex	Female	19052(66.5)	317(1.7)	18735(98.3)	< 0.001	5877(51.6)	88(1.5)	5789(98.5)	< 0.001
	Male	9605(33.5)	338(3.5)	9267(96.5)		5507(48.4)	168(3.1)	5339(96.9)	
Age		44.16±19.61	38.24±15.97	44.30±19.66	< 0.001	5.69±5.42	10.72±3.97	5.57±5.39	< 0.001
Ethnicity	Hispanic or Latino	4769(16.6)	137(2.9)	4632(97.1)	0.003	3494(30.7)	85(2.4)	3409(97.6)	0.378
	Not Hispanic or Latino	23888(83.4)	518(2.2)	23370(97.8)		7890(69.3)	171(2.2)	7719(97.8)	
Race	White	21993(76.7)	550(2.5)	21443(97.5)	< 0.001	8213(72.1)	225(2.7)	7988(97.3)	< 0.001
	Black/African American	5577(19.5)	62(1.1)	5515(98.9)		2590(22.8)	20(0.8)	2570(99.2)	
	Asian	617(2.2)	27(4.4)	590(95.6)		304(2.7)	6(2.0)	298(98.0)	
	Native Hawaiian/Other Pacific Islander	171(0.6)	6(3.5)	165(96.5)		107(0.9)	2(1.9)	105(98.1)	
	American Indian/Alaska Native	188(0.7)	8(4.3)	180(95.7)		88(0.8)	1(1.1)	87(98.9)	
	More than one race reported	111(0.4)	2(1.8)	109(98.2)		82(0.7)	2(2.4)	80(97.6)	
Residence	Private residence	27749(96.8)	644(2.3)	27105(97.7)	0.004	11318(99.4)	254(2.2)	11064(97.8)	0.646
	Nursing home	466(1.6)	1(0.2)	465(99.8)		15(0.1)	0(0.0)	15(100.0)	
	Homeless/homeless shelter	134(0.5)	0(0.0)	134(100.0)		8(0.1)	0(0.0)	8(100.0)	
	Other	308(1.1)	10(3.2)	298(96.8)		43(0.4)	2(4.7)	41(95.3)	
Insurance	Private insurance	9942(34.7)	372(3.7)	9570(96.3)	< 0.001	3342(29.4)	122(3.7)	3220(96.3)	< 0.001
	Medicare	6351(22.2)	64(1)	6287(99.0)		147(1.3)	2(1.4)	145(98.6)	
	Medicaid or CHIP or other state-based program	7494(26.2)	111(1.5)	7383(98.5)		6990(61.4)	110(1.6)	6880(98.4)	
	Worker’s compensation	41(0.1)	2(4.9)	39(95.1)		2(0.0)	0(0.0)	2(100.0)	
	Self-pay	3818(13.3)	87(2.3)	3731(97.7)		666(5.9)	15(2.3)	651(97.7)	
	No charge/Charity	312(1.1)	9(2.9)	303(97.1)		26(0.2)	0(0.0)	26(100.0)	
	Other	699(2.4)	10(1.4)	689(98.6)		211(1.9)	7(3.3)	204(96.7)	
Visit year	2005	1988(6.9)	69(3.5)	1919(96.5)	< 0.001	538(4.7)	31(5.8)	507(94.2)	< 0.001
	2006	2079(7.3)	67(3.2)	2012(96.8)		767(6.7)	28(3.7)	739(96.3)	
	2007	2248(7.8)	67(3.0)	2181(97.0)		593(5.2)	18(3.0)	575(97.0)	
	2008	2244(7.8)	58(2.6)	2186(97.4)		594(5.2)	23(3.9)	571(96.1)	
	2009	2452(8.6)	56(2.3)	2396(97.7)		1270(11.2)	27(2.1)	1243(97.9)	
	2010	2716(9.5)	64(2.4)	2652(97.6)		1231(10.8)	13(1.1)	1218(98.9)	
	2011	2569(9.0)	71(2.8)	2498(97.2)		1038(9.1)	23(2.2)	1015(97.8)	
	2012	2543(8.9)	55(2.2)	2488(97.8)		1025(9.0)	22(2.1)	1003(97.9)	
	2013	2200(7.7)	28(1.3)	2172(98.7)		911(8.0)	20(2.2)	891(97.8)	
	2014	2207(7.7)	37(1.7)	2170(98.3)		1094(9.6)	17(1.6)	1077(98.4)	
	2015	1942(6.8)	27(1.4)	1915(98.6)		826(7.3)	15(1.8)	811(98.2)	
	2016	1870(6.5)	34(1.8)	1836(98.2)		782(6.9)	6(0.8)	776(99.2)	
	2017	1599(5.6)	22(1.4)	1577(98.6)		715(6.3)	13(1.8)	702(98.2)	
Visit month	January	2464(8.6)	59(2.4)	2405(97.6)	0.988	1058(9.3)	22(2.1)	1036(97.9)	0.009
	February	2181(7.6)	48(2.2)	2133(97.8)		1018(8.9)	20(2.0)	998(98.0)	
	March	2408(8.4)	56(2.3)	2352(97.7)		987(8.7)	16(1.6)	971(98.4)	
	April	2432(8.5)	51(2.1)	2381(97.9)		966(8.5)	16(1.7)	950(98.3)	
	May	2480(8.7)	58(2.3)	2422(97.7)		1006(8.8)	22(2.2)	984(97.8)	
	June	2385(8.3)	57(2.4)	2328(97.6)		892(7.8)	17(1.9)	875(98.1)	
	July	2468(8.6)	61(2.5)	2407(97.5)		841(7.4)	16(1.9)	825(98.1)	
	August	2641(9.2)	60(2.3)	2581(97.7)		893(7.8)	22(2.5)	871(97.5)	
	September	2394(8.4)	58(2.4)	2336(97.6)		958(8.4)	38(4.0)	920(96.0)	
	October	2167(7.6)	50(2.3)	2117(97.7)		890(7.8)	31(3.5)	859(96.5)	
	November	2402(8.4)	45(1.9)	2357(98.1)		976(8.6)	18(1.8)	958(98.2)	
	December	2235(7.8)	52(2.3)	2183(97.7)		899(7.9)	18(2.0)	881(98.0)	
Visit day	Sunday	3926(13.7)	83(2.1)	3843(97.9)	0.469	1812(15.9)	38(2.1)	1774(97.9)	0.451
	Monday	4498(15.7)	90(2.0)	4408(98.0)		1808(15.9)	37(2.0)	1771(98.0)	
	Tuesday	4229(14.8)	107(2.5)	4122(97.5)		1637(14.4)	29(1.8)	1608(98.2)	
	Wednesday	4201(14.7)	102(2.4)	4099(97.6)		1508(13.2)	44(2.9)	1464(97.1)	
	Thursday	4081(14.2)	91(2.2)	3990(97.8)		1558(13.7)	39(2.5)	1519(97.5)	
	Friday	3879(13.5)	100(2.6)	3779(97.4)		1460(12.8)	33(2.3)	1427(97.7)	
	Saturday	3843(13.4)	82(2.1)	3761(97.9)		1601(14.1)	36(2.2)	1565(97.8)	
Arrival time	Morning	7966(27.8)	195(2.4)	7771(97.6)	0.697	2435(21.4)	53(2.2)	2382(97.8)	0.136
	Afternoon	8027(28.0)	178(2.2)	7849(97.8)		2605(22.9)	74(2.8)	2531(97.2)	
	Evening	6111(21.3)	133(2.2)	5978(97.8)		2993(26.3)	61(2.0)	2932(98.0)	
	Night	6553(22.9)	149(2.3)	6404(97.7)		3351(29.4)	68(2.0)	3283(98.0)	
Temperature		36.78±0.59	36.92±0.64	36.77±0.58	< 0.001	37.44±1.06	37.18±0.83	37.45±1.06	< 0.001
Triage level	Immediate	375(1.3)	21(5.6)	354(94.4)	< 0.001	80(0.7)	8(10.0)	72(90.0)	< 0.001
	Emergent	2353(8.2)	82(3.5)	2271(96.5)		607(5.3)	32(5.3)	575(94.7)	
	Urgent	20269(70.7)	483(2.4)	19786(97.6)		5854(51.4)	170(2.9)	5684(97.1)	
	Semi-urgent	4677(16.3)	57(1.2)	4620(98.8)		4166(36.6)	38(0.9)	4128(99.1)	
	Nonurgent	983(3.4)	12(1.2)	971(98.8)		677(5.9)	8(1.2)	669(98.8)	
Is injury/poisoning intentional	Intentional	145(0.5)	2(1.4)	143(98.6)	< 0.001	19(0.2)	0(0.0)	19(100)	0.007
	Unintentional	2113(7.4)	12(0.6)	2101(99.4)		480(4.2)	1(0.2)	479(99.8)	
	Questionable injury status	26399(92.1)	641(2.4)	25758(97.6)		10885(95.6)	255(2.3)	10630(97.7)	
Visit related to an injury/poison/adverse effect of medical treatment with in 72 hours	No	26123(91.2)	644(2.5)	25479(97.5)	< 0.001	10831(95.1)	256(2.4)	10575(97.6)	0.001
	Yes	2534(8.9)	11(0.5)	2523(99.5)		553(4.9)	0(0.0)	553(100.0)	
Systolic blood pressure		134.17±22.87	130.93±19.10	134.24±22.94	0.190	110.53±14.20	117.16±15.25	110.38±14.14	0.003
Diastolic blood pressure		78.62±18.28	76.76±12.11	78.67±18.40	< 0.001	69.16±49.87	67.43±12.34	69.20±50.40	< 0.001
Pulse Oximetry		87.66±17.99	87.38±19.08	87.66±17.96	< 0.001	120.70±31.04	103.63±23.95	121.10±31.08	< 0.001
72h Revisit	Yes	1394(4.9)	22(1.6)	1372(98.4)	0.070	448(3.9)	10(2.2)	438(97.8)	0.981
	No	27263(95.1)	633(2.3)	26630(97.7)		10936(96.1)	246(2.2)	10690(97.8)	
Pain level	Mild	5711(19.9)	62(1.1)	5649(98.9)	< 0.001	6337(55.7)	36(0.6)	6301(99.4)	< 0.001
	Moderate	9496(33.1)	226(2.4)	9270(97.6)		3210(28.2)	116(3.6)	3094(96.4)	
	Very severe	13450(46.9)	367(2.7)	13083(97.3)		1837(16.1)	104(5.7)	1733(94.3)	
Diagnostic services provided	No	3665(12.8)	47(1.3)	3618(98.7)	< 0.001	3944(34.6)	21(0.5)	3923(99.5)	< 0.001
	Yes	24992(87.2)	608(2.4)	24384(97.6)		7440(65.4)	235(3.2)	7205(96.8)	

Missing value for patient’s residence type, diagnostic services provided, arrival time, body temperature and whether the visit is related to injury/poisoning is lower than 5%. Missing values for source of payment, pulse oximetry are between 5 and 10%. Missing value for race, heart rate, 72 h revisit, systolic and diastolic blood pressure are between 10 and 15%. Missing value for ethnicity and triage level are 15 and 20%. Missing value for pain level is 24.89%. Missing value for is injury/poisoning intentional is 43.20%

*Note*: ^1^*p*-values in this table came from the chi-squared test for categorical variables and from the t-test for continuous variables

The crude and adjusted odds ratio of adult and pediatric ED patients with acute appendicitis (vs. non-appendicitis) for each predictive factor using binary LR are presented in Table 2. The adjusted analysis showed that the risk of being diagnosed with AA was higher in adult males (aOR=2.327; 95% CI:1.984-2.728) and pediatric males (aOR=2.759; 95% CI:2.102-3.622) than females. Compared with patients with private insurance, adults (aOR=0.462; 95%CI: 0.370-0.578) and pediatric patients (aOR=0.691; 95% CI: 0.517-0.923) with Medicaid or Children's Health Insurance Program (CHIP) or other state-based program had a lower risk of being diagnosed with AA. Adults and pediatric patients with immediate triage levels were more likely to be diagnosed with AA. The risk of adults with moderate (aOR=2.016; 95% CI: 1.513-2.687) and very severe (aOR=2.527; 95% CI: 1.915-3.335) pain levels had greater odds than those being diagnosed with AA with mild pain. Similarly, the risk of pediatric patients with moderate (aOR=5.291; 95% CI: 3.587-7.805) and very severe (aOR=8.094; 95% CI: 5.414-12.099) pain levels had greater odds than those being diagnosed with AA with mild pain. Adults (aOR = 2.268; 95% CI: 1.445-3.560) and pediatric patients (aOR = 3.385; 95% CI: 2.106-5.441) who received diagnostic services had greater odds of AA than those who did not receive diagnostic services.

**Table 2:** Adjusted odds ratio (aOR) of characteristics of adult and pediatric during the emergency department visit (appendicitis vs. non-appendicitis), NHAMCS 2005–2017

		Adult		Pediatric	
		Crude	Adjusted	Crude	Adjusted
Sex	Famale	Reference	Reference	Reference	Reference
	Male	2.156(1.846-2.518)	2.327(1.984-2.728)	2.070(1.595-2.686)	2.759(2.102-3.622)
Ethnicity	Hispanic or Latino	Reference	Reference	Reference	Reference
	Not Hispanic or Latino	0.749(0.619-0.907)	0.722(0.590-0.884)	0.888(0.683-1.156)	0.830(0.618-1.116)
Race	White	Reference	Reference	Reference	Reference
	Black/African American	0.438(0.337-0.571)	0.502(0.382-0.659)	0.276(0.175-0.437)	0.340(0.210-0.552)
	Asian	1.784(1.202-2.648)	1.679(1.117-2.522)	0.715(0.315-1.621)	0.856(0.366-2.002)
	Native Hawaiian/Other Pacific Islander	1.418(0.625-3.216)	1.442(0.627-3.314)	0.676(0.166-2.757)	0.704(0.159-3.124)
	American Indian/Alaska Native	1.733(0.849-3.536)	1.853(0.888-3.863)	0.408(0.057-2.943)	0.508(0.068-3.816)
	More than one race reported	0.715(0.176-2.904)	0.623(0.151-2.566)	0.888(0.217-3.633)	1.268(0.286-5.610)
Residence	Private residence	Reference	Reference	Reference	Reference
	Nursing home	0.091(0.013-0.645)	0.182(0.025-1.312)	-	-
	Homeless/homeless shelter	-	-	-	-
	Other	1.412(0.749-2.665)	1.646(0.856-3.165)	2.125(0.511-8.832)	1.690(0.373-7.664)
Insurance	Private insurance	Reference	Reference	Reference	Reference
	Medicare	0.262(0.200-0.342)	0.297(0.226-0.390)	0.364(0.089-1.487)	0.451(0.106-1.910)
	Medicaid or CHIP or other state-based program	0.387(0.312-0.479)	0.462(0.370-0.578)	0.422(0.325-0.548)	0.691(0.517-0.923)
	Worker’s compensation	1.319(0.317-5.484)	1.926(0.438-8.466)	-	-
	Self-pay	0.600(0.474-0.760)	0.574(0.450-0.732)	0.608(0.353-1.047)	0.611(0.345-1.082)
	No charge/Charity	0.764(0.391-1.495)	0.755(0.381-1.495)	-	-
	Other	0.373(0.198-0.703)	0.378(0.199-0.717)	0.906(0.417-1.965)	1.142(0.489-2.667)
Visit year	2005	Reference	Reference	Reference	Reference
	2006	0.926(0.658-1.304)	0.857(0.604-1.215)	0.620(0.367-1.046)	0.813(0.464-1.425)
	2007	0.854(0.607-1.202)	0.811(0.572-1.150)	0.512(0.283-0.926)	0.572(0.305-1.074)
	2008	0.738(0.518-1.052)	0.730(0.508-1.048)	0.659(0.379-1.145)	0.701(0.388-1.265)
	2009	0.650(0.455-0.929)	0.617(0.428-0.890)	0.355(0.210-0.601)	0.524(0.299-0.921)
	2010	0.671(0.475-0.948)	0.633(0.443-0.903)	0.175(0.091-0.336)	0.253(0.127-0.502)
	2011	0.790(0.565-1.107)	0.759(0.537-1.073)	0.371(0.214-0.642)	0.546(0.303-0.982)
	2012	0.615(0.429-0.881)	0.598(0.413-0.866)	0.359(0.206-0.626)	0.432(0.238-0.784)
	2013	0.359(0.230-0.559)	0.372(0.236-0.585)	0.367(0.207-0.651)	0.548(0.296-1.014)
	2014	0.474(0.317-0.710)	0.475(0.314-0.719)	0.258(0.142-0.471)	0.385(0.203-0.729)
	2015	0.392(0.250-0.615)	0.395(0.249-0.625)	0.302(0.162-0.566)	0.408(0.209-0.796)
	2016	0.515(0.340-0.780)	1.076(0.601-1.927)	0.126(0.052-0.305)	0.363(0.142-0.929)
	2017	0.388(0.239-0.630)	0.406(0.248-0.665)	0.303(0.157-0.585)	0.389(0.193-0.781)
Visit month	January	Reference	Reference	Reference	Reference
	February	0.917(0.624-1.349)	0.876(0.592-1.295)	0.944(0.512-1.740)	0.916(0.483-1.735)
	March	0.971(0.670-1.405)	0.911(0.625-1.328)	0.776(0.405-1.486)	0.891(0.455-1.743)
	April	0.873(0.598-1.275)	0.822(0.559-1.209)	0.793(0.414-1.519)	0.868(0.444-1.699)
	May	0.976(0.676-1.409)	0.936(0.644-1.359)	1.053(0.579-1.913)	1.206(0.648-2.242)
	June	0.998(0.690-1.443)	0.935(0.643-1.360)	0.915(0.483-1.734)	1.064(0.548-2.067)
	July	1.033(0.719-1.484)	0.985(0.681-1.424)	0.913(0.477-1.750)	0.987(0.501-1.943)
	August	0.948(0.659-1.363)	0.877(0.606-1.271)	1.189(0.654-2.162)	1.204(0.644-2.249)
	September	1.012(0.701-1.461)	0.969(0.666-1.408)	1.945(1.142-3.313)	2.33(1.331-4.082)
	October	0.963(0.658-1.410)	0.879(0.597-1.295)	1.699(0.977-2.957)	1.834(1.024-3.286)
	November	0.778(0.526-1.152)	0.769(0.517-1.145)	0.885(0.472-1.660)	1.063(0.552-2.044)
	December	0.971(0.666-1.416)	0.950(0.648-1.395)	0.962(0.513-1.805)	1.244(0.645-2.400)
Visit day	Sunday	Reference	Reference	Reference	Reference
	Monday	0.945(0.699-1.278)	0.954(0.703-1.295)	0.975(0.617-1.541)	0.903(0.559-1.458)
	Tuesday	1.202(0.899-1.606)	1.222(0.910-1.640)	0.842(0.517-1.372)	0.842(0.507-1.400)
	Wednesday	1.152(0.859-1.544)	1.163(0.864-1.566)	1.403(0.904-2.178)	1.370(0.862-2.178)
	Thursday	1.056(0.782-1.427)	1.086(0.801-1.474)	1.199(0.763-1.884)	1.182(0.735-1.900)
	Friday	1.225(0.913-1.645)	1.300(0.963-1.753)	1.080(0.674-1.730)	1.086(0.662-1.781)
	Saturday	1.009(0.742-1.374)	1.032(0.755-1.411)	1.074(0.677-1.703)	0.987(0.608-1.603)
Arrival time	Morning	Reference	Reference	Reference	Reference
	Afternoon	0.904(0.736-1.110)	0.953(0.774-1.175)	1.314(0.919-1.878)	1.355(0.931-1.972)
	Evening	0.887(0.709-1.108)	0.900(0.718-1.129)	0.935(0.645-1.356)	0.956(0.648-1.409)
	Night	0.927(0.747-1.151)	0.872(0.700-1.086)	0.931(0.648-1.338)	0.911(0.624-1.332)
Triage level	Immediate	Reference	Reference	Reference	Reference
	Emergent	0.609(0.372-0.996)	0.596(0.358-0.990)	0.501(0.222-1.129)	0.671(0.267-1.684)
	Urgent	0.412(0.263-0.645)	0.447(0.279-0.715)	0.269(0.128-0.568)	0.346(0.146-0.820)
	Semi-urgent	0.208(0.125-0.347)	0.234(0.138-0.398)	0.083(0.037-0.184)	0.176(0.071-0.439)
	Nonurgent	0.208(0.101-0.428)	0.213(0.103-0.444)	0.108(0.039-0.295)	0.213(0.070-0.644)
Is injury/poisoning intentional	Intentional	Reference	Reference	Reference	Reference
	Unintentional	0.408(0.091-1.842)	0.293(0.062-1.395)	-	-
	Questionable injury status	1.779(0.440-7.199)	0.216(0.037-1.249)	-	-
Visit related to an injury/poison/adverse effect of medical treatment within 72 hours	No	Reference	Reference	Reference	Reference
	Yes	0.176(0.097-0.320)	0.119(0.042-0.339)	-	-
72h Revisit	Yes	Reference	Reference	Reference	Reference
	No	1.482(0.966-2.275)	1.374(0.890-2.122)	1.008(0.532-1.910)	0.893(0.458-1.740)
Pain level	Mild	Reference	Reference	Reference	Reference
	Moderate	2.221(1.674-2.948)	2.016(1.513-2.687)	6.562(4.504-9.561)	5.291(3.587-7.805)
	Very severe	2.556(1.949-3.351)	2.527(1.915-3.335)	10.504(7.164-15.401)	8.094(5.414-12.099)
Diagnostic services provided	No	Reference	Reference	Reference	Reference
	Yes	1.919(1.424-2.588)	2.268(1.445-3.560)	6.093(3.893-9.538)	3.385(2.106-5.441)

In Fig. S1, before using the LR and RF approaches, we showed the contribution (weights) of each 128 Doc2Vec output to the first 24 principle components for the unstructured data.

**Fig. 1:**
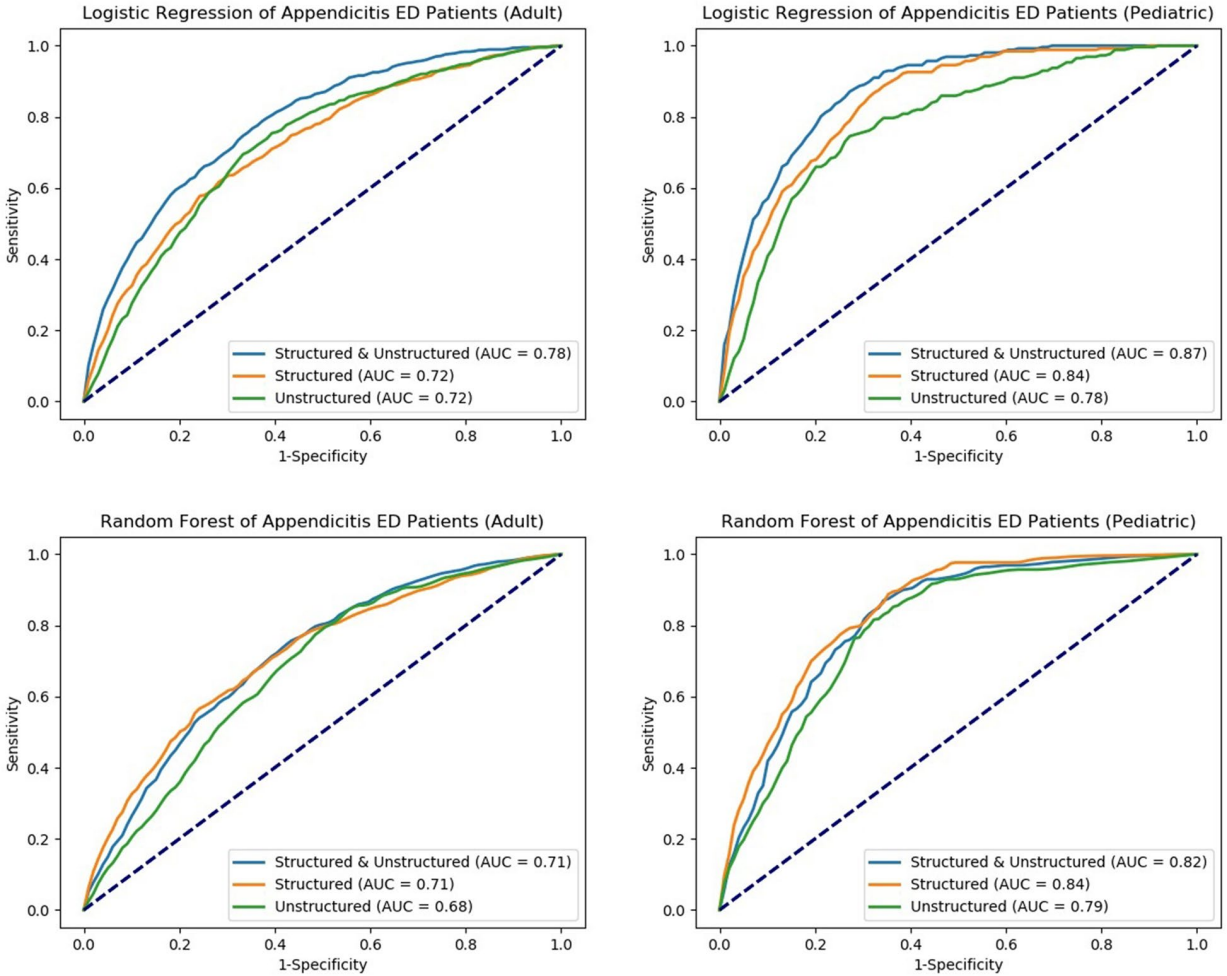
ROC curves for the LR and RF models for predicting the diagnosed appendicitis (adult and pediatric)

As shown in Table 3 and Fig. 1, for the LR model identifying AA diagnosis among adult ED patients, the AUC was 0.72 (95% CI: 0.69–0.75) for structured variables only, and 0.72 (95% CI: 0.69–0.75) for unstructured variables only, and 0.78 (95% CI: 0.76–0.80) when including both structured and unstructured variables. For the LR model identifying AA diagnosis among pediatric ED patients, the AUC was 0.84 (95% CI: 0.79–0.89) for structured variables only, 0.78 (95% CI: 0.72–0.84) for unstructured variables, and 0.87 (95% CI: 0.83–0.91) when including both structured and unstructured variables.

**Table 3:** Predictive performance of LR and RF models with 5-fold classification in identifying diagnosed appendicitis ED patients, NHAMCS 2005-2017

Models	Sensitivity (95% CI)	Specificity (95% CI)	Threshold (95% CI)	Accuracy (95% CI)	AUC (95% CI)
**LR for adult**					
Structured + Unstructured variables	0.73 (0.68-0.78)	0.68 (0.59-0.77)	0.12 (0.10-0.14)	0.96 (0.95-0.97)	0.78 (0.76-0.80)
Structured variables	0.64 (0.54-0.74)	0.70 (0.60-0.80)	0.08 (0.06-0.10)	0.95 (0.93-0.97)	0.72 (0.69-0.75)
Unstructured variables	0.69 (0.64-0.74)	0.67 (0.63-0.71)	0.05 (0.05-0.05)	0.93 (0.92-0.94)	0.72 (0.69-0.75)
**LR for pediatric**					
Structured + Unstructured variables	0.81 (0.74-0.88)	0.78 (0.73-0.83)	0.13 (0.09-0.17)	0.95 (0.93-0.97)	0.87 (0.83-0.91)
Structured variables	0.83 (0.71-0.95)	0.71 (0.59-0.83)	0.11 (0.04-0.18)	0.94 (0.89-0.99)	0.84 (0.79-0.89)
Unstructured variables	0.75 (0.70-0.80)	0.73 (0.61-0.85)	0.06 (0.04-0.08)	0.90 (0.83-0.97)	0.78 (0.72-0.84)
**RF for adult**					
Structured + Unstructured variables	0.67 (0.56-0.78)	0.71 (0.59-0.83)	0.13 (0.11-0.15)	0.97 (0.96-0.98)	0.75 (0.71-0.79)
Structured variables	0.68 (0.59-0.77)	0.65 (0.58-0.72)	0.14 (0.13-0.15)	0.97 (0.97-0.97)	0.71 (0.65-0.77)
Unstructured variables	0.65 (0.59-0.71)	0.63 (0.55-0.71)	0.11 (0.10-0.12)	0.96 (0.95-0.97)	0.68 (0.64-0.72)
**RF for pediatric**					
Structured + Unstructured variables	0.82 (0.73-0.91)	0.75 (0.69-0.81)	0.13 (0.12-0.14)	0.96 (0.96-0.96)	0.86 (0.84-0.88)
Structured variables	0.81 (0.75-0.87)	0.72 (0.63-0.81)	0.15 (0.12-0.18)	0.96 (0.95-0.97)	0.84 (0.79-0.89)
Unstructured variables	0.8 (0.71-0.89)	0.65 (0.59-0.71)	0.11 (0.09-0.13)	0.95 (0.94-0.96)	0.78 (0.76-0.80)

For the RF model identifying AA diagnosis among adult ED patients, the AUC was 0.71 (95% CI: 0.65–0.77) for structured variables, 0.68 (95% CI: 0.64–0.72) for unstructured variables, and 0.75 (95% CI: 0.71–0.79) for structured and unstructured variables. For the RF model identifying AA diagnosis among pediatric ED patients, the AUC was 0.84 (95% CI: 0.83–0.85) for structured variables, 0.78 (95% CI: 0.76–0.80) for unstructured variables, and 0.86 (95% CI: 0.84–0.88) for structured and unstructured variables. The discrimination ability of different models, as represented by ROC curves, is shown in Fig. 1.

The standardized and non-standardized coefficients of structured variables were used as modeling examples (Tables S3 and S4) to determine whether to diagnose AA among adult and pediatric ED patients. The standardized coefficient can be used to compare which variable has the greater influence on the prediction of confirmed AA. The standardized coefficients of insurance and triage levels were highest among adults with ED. Among children with ED, the highest standardized coefficients were insurance and pain levels.

## Discussion

In this study, we used data from the 2005-2017 NHAMCS ED survey and applied statistical models to predict whether adult and pediatric patients were diagnosed with AA. A novel part of this study was a traditional statistics and ML approach (LR algorithm) and a advanced machine learning modeling techniques (RF algorithm), which can be used to diagnose and identify the clinical problem of appendicitis and to judge the predicted performance of the two machine learning modeling techniques through a series of indicators. In addition, in the aspect of preprocessing of unstructured text information, we used Doc2Vec technology in natural language processing to extract features of unstructured text and use it for modeling and prediction, so as to improve the prediction ability of the two machine learning models. In general, the performance of both models was significantly improved after NLP by using predictors that combined structured data with unstructured data.

To our knowledge, this is the first time that Doc2Vec technology of NLP has been used to conduct unstructured text analysis of the reason for patient visit and the reason for injury to predict AA diagnosis using NHAMCS ED survey data. This study also serves as a teaching case to help physicians, nurses, researchers, and others learn about NLP technologies. Combined with the structured data, LR algorithm and RF algorithm were used to establish the diagnosis and prediction model of emergency hospitalized appendicitis. Many other studies have shown that in the fields of electronic case mining and bioinformatics, the predictive performance of models can be greatly improved by incorporating textual information [34–37]. There are several potential explanations for the incremental gains in the prediction ability by the NLP. First, NLP can more effectively capture more word and context information from the unstructured text, which cannot be addressed by traditional text analysis approaches, such as word spotting and manual rules [38]. Additionally, end-to-end training and learning of representations differentiate deep learning from traditional ML methods and make it a powerful tool for NLP [39]. Moreover, Doc2Vec technology allows us to extract/infer specific features for both the word and the paragraph, which cannot be solved by word2vec technology. Our results show that the value of AUC is the highest when both structured and unstructured data are included in the prediction model.

Although many previous studies have shown that the performance of a RF algorithm is better than that of a LR algorithm [40–42], LR and RF algorithms were used for different patients in our study, and the results showed that the predictive performance of LR algorithm was no different from the RF algorithm for both adult and pediatric patients. This may be because LR model works well as a classifier if the relationship between the input variables (structured variables) and output variable (AA) is linear and the data is relatively balanced between classes. If the relationship between the input and the output variable is linear, RF algorithm will only approximate linear regression methods like LR in the limit case of an infinite number of trees. RF algorithm exchanges a high degree of variance between each tree for a low bias in predicting the outcome variable. A more unbiased estimate may be given if other methods are assumed not to violate the linearity, collinearity, and homogeneity of the parameters [43–45].

Compared with ED patients with private insurance, patients with Medicaid or CHIP or other state-based programs and self-pay patients had a significantly lower risk of being diagnosed with appendicitis. The reasons for these differences should be further explored in future studies to determine the appropriateness of including or excluding these variables in predictive models, which is important to determine whether such predictive models can be used as a more objective tool to predict whether a patient has appendicitis based on the clinical context [46]. Sex, race, ethnicity, triage level, pain level and diagnostic services provided were also found to be important predictors for identifying patients with appendicitis. As expected, patients with immediate triage level were more likely to be diagnosed with appendicitis than those with other triage levels. Patients with moderate and very severe pain levels were generally more likely to be diagnosed with AA than those with mild pain levels.

The clinical practice of adult ED is quite different from that of pediatric ED. In particular, the diagnosis of appendicitis in pediatric populations is more complex and time-consuming than that in adults because of their physiological and developmental differences [47]. Compared with patients with immediate triage level, the risk of diagnosis of urgent, semi-urgent and nonurgent appendicitis in pediatric patients is lower than adult patients. However, compared with mild patients, pediatric patients with moderate pain levels and very severe AA had a higher risk of diagnosis than adults.

Since the prediction model is based on whether patients with ED will eventually be diagnosed with AA, the prediction model can not only predict AA, but also help doctors, nurses and triage personnel to choose more helpful examination items in advance, so as to make more efficient use of medical resources. Previous studies have shown that because the ED is a critical staging area for critically ill patients, developing more efficient tools to avoid overcrowding and increase the efficiency of the use of healthcare resources in the ED and ultimately improve the quality of care and health outcomes for ED patients [48–50]. The prediction model developed in our study for adults and pediatric ED patients with diagnosed appendicitis is consistent with the goal of establishing a better decision system in ED [51, 52].

The prediction model of diagnosed ED patients with AA produced in this study is designed to help doctors, nurses, and triage personnel make decisions and cannot completely replace their roles. Although we developed an improved prediction model of diagnosing ED patients with AA, it still needs the actual clinical work. There is a certain risk that the model is still imperfect at present, so it may increase the possibility of misdiagnosis of AA if clinicians rely on it more than as an assistive tool.

## Limitations

Our study has several limitations. First, due to the large span of survey years, the questionnaire variables are inconsistent in different years, so some available variables are not included in the prediction model, such as complications, arriving by ambulance, etc., which may affect the prediction ability of the model [53]. Second, the NHAMCS data did not gather more useful clinical variables for the diagnosis of appendicitis, such as hyperbilirubinemia, white blood cells (WBCs) count and absence of inflammatory changes, etc. However, the goal of this study is not to use a large number of predictors to build predictive models, but to use a limited number of predictors to build machine learning models, which are often easier to practice. However, the results of this study still lack clinical operability and need to be further verified and improved. Third, more dimensions of the feature extraction technology of Doc2Vec were not attempted. The dimension values used in this paper were mainly based on the experience of previous literature, which may affect the prediction ability of the prediction model [38, 54]. Fourth, The dataset is a large administrative dataset that may have more limitations such as the sampling techniques used to generate the data, the decreasing number of AA as the years go by, and the lack of clinical context of the patients that only come from using more robust clinical data [55, 56]. Finally, The low incidence of AA in the study population suggests that the number of patients actually considered or AA was much smaller than the inclusion criteria suggest. Only 2-3% positive is very low as compared to other studies, which may affect the predictive performance of the model.

## Conclusions

Based on the analysis of 40,041 patients with AA-related symptoms in the NHAMCS ED survey, we examined the information relating to the patients’ social economic, demographic and clinical factors during the patients’ ED visits, including the unstructured free-text, such as the reason for visits and the cause of the injury, and developed a prediction model to diagnose AA for adults and children. Although external prospective validation is necessary, these observations suggest an opportunity to apply advanced predictive methods to routinely available triage data -- as an assistive technique -- to enhance clinicians’ diagnostic decisions, which in turn will lead to more accurate and effective clinical identification of AA in the ED.

## Supplementary Information

**Additional file 1: Table S1.** Diagnosis and Procedure Codes. **Table S2.** Sample size of Diagnosis and Procedure Codes between 2005 to 2017. **Table S3.** Parameter estimation with structured variables of the logistic regression for adult ED patients, NHAMCS 2005-2017. **Table S4.** Parameter estimation with structured variables of the logistic regression for pediatric ED patients, NHAMCS 2005-2017. **Figure S1.** The contribution (weights) of each 128 Doc2Vec output to the first 24 principle components

## Data Availability

The datasets and code generated during and/or analysed during the current study are available from the corresponding author on reasonable request.

## Abbreviations

AA: Acute Appendicitis
NHAMCS: National Hospital Ambulatory Medical Care Survey
ED: Emergency Department
NLP: Natural Language Processing
ICD: International Classification of Diseases
ML: Machine Learning
EHRs: Electronic Health Records
CDC: Centers for Disease Control and Prevention
ICD-9-CM: ICD, 9th Revision, Clinical Modification
ICD-10-CM: ICD, 10th Revision, Clinical Modification
GEM: General Equivalence Mapping
NCHS: National Center for Health Statistics
k-NN: k-Nearest Neighbors
AI: Artificial Intelligence
LR: Logistic Regression
GLM: General Linear Model
MLE: Maximum Likelihood Estimation
RF: Random Forests
PCA: Principal Component Analysis
CHIP: Children’s Health Insurance Program
AUC: Area Under the Receiver Operating Curve
ROC: Receiver Operating Characteristics
FPR: False Positive Rate
TPR: True Positive Rate
OR: Odds Ratio
CI: Confidence Interval
WBCs: White Blood Cells
