# Supplementary material for: Prediction of acute appendicitis among patients with undifferentiated abdominal pain at emergency department
Source: BMC Med Res Methodol. 2022 Jan 14;22:18. doi: 10.1186/s12874-021-01490-9 (PMC8759254; doi:10.1186/s12874-021-01490-9)
Supplement: Supplementary file 1 — Additional file 1: Table S1. Diagnosis and Procedure Codes. Table S2. Sample size of Diagnosis and Procedure Codes between 2005 to 2017. Table S3. Parameter estimation with structured variables of the logistic regression for adult ED patients, NHAMCS 2005-2017. Table S4. Parameter estimation with structured variables of the logistic regression for pediatric ED patients, NHAMCS 2005-2017. Figure S1. The contribution (weights) of each 128 Doc2Vec output to the first 24 principle components [file 12874_2021_1490_MOESM1_ESM.docx]

| **Table S1. Diagnosis and Procedure Codes** | | | | |
| --- | --- | --- | --- | --- |
|  | **ICD-9-CM** | **ICD-10-CM** | **DATASET 2005-2014 （ICD-9-CM）** | **DATASET 2015-2017 （ICD-10-CM）** |
| **Disease** |  |  |  |  |
| Appendicitis | 540 | K35 | 5400- 5401- 5409- | K35- K352 K353 K358 |
|  | 541 | K36 | 541-- | K36- |
|  | 542 | K37 | 542-- | K37- |
| **Symptoms** |  |  |  |  |
| Abdominal pain | 789 | R10 | 78900 78901 78902 78903 78904 78905 78906 78907 78909 | R100 R101 R102 R103 R108 R109 |
|  |  |  |  |  |
| Vomiting/nausea | 787 | R11 | 78701 78702 78703 78704 | R110 R111 R112 |
|  |  |  |  |  |
| Diarrhea | 787.91 | R19.7 | 78791 | R197 |
| Constipation | 564 | K59.0 | 56400 56401 56402 56409 | K59- (K590) |
|  |  |  |  |  |
| Fever | 780.6 | R50.9 | 78060 | R509 |

**Table S2. Sample size of Diagnosis and Procedure Codes between 2005 to 2017**

|  | **2005** | **2006** | **2007** | **2008** | **2009** | **2010** | **2011** | **2012** | **2013** | **2014** | **2015** | **2016** | **2017** | **Total** |
| --- | --- | --- | --- | --- | --- | --- | --- | --- | --- | --- | --- | --- | --- | --- |
| Appendicitis |  |  |  |  |  |  |  |  |  |  |  |  |  |  |
|  | 100 | 95 | 85 | 81 | 83 | 77 | 94 | 77 | 48 | 54 | 42 | 40 | 35 | 911 |
|  |  |  |  |  |  |  |  |  |  |  |  |  |  |  |
| Abdominal pain | 2426 | 2751 | 2756 | 2757 | 3639 | 3870 | 3513 | 3491 | 3063 | 3247 | 2726 | 2612 | 2279 | 39130 |
| Vomiting/nausea |  |  |  |  |  |  |  |  |  |  |  |  |  |  |
| Diarrhea |  |  |  |  |  |  |  |  |  |  |  |  |  |  |
| Constipation |  |  |  |  |  |  |  |  |  |  |  |  |  |  |
| Fever |  |  |  |  |  |  |  |  |  |  |  |  |  |  |
| Total | 2526 | 2846 | 2841 | 2838 | 3722 | 3947 | 3607 | 3568 | 3111 | 3301 | 2768 | 2652 | 2314 | 40041 |

| **Table S3. Parameter estimation with structured variables of the logistic regression for adult ED patients, NHAMCS 2005-2017** | | | | | |
| --- | --- | --- | --- | --- | --- |
|  | | **Coefficient** | **Standard Error** | **Standardized Coefficient** | ***p* value** |
| Sex | Famale | Reference |  |  |  |
|  | Male | 0.920 | 0.083 | 0.239 | <0.001 |
| Age |  | -0.017 | 0.003 | -0.182 | <0.001 |
| Ethnicity | Hispanic or Latino | Reference |  |  |  |
|  | Not Hispanic or Latino | -0.269 | 0.104 | -0.055 | 0.010 |
| Race | White | Reference |  |  |  |
|  | Black/African American | -0.755 | 0.140 | -0.278 | <0.001 |
|  | Asian | 0.476 | 0.209 | 0.175 | 0.023 |
|  | Native Hawaiian/Other Pacific Islander | 0.271 | 0.429 | 0.100 | 0.528 |
|  | American Indian/Alaska Native | 0.556 | 0.378 | 0.204 | 0.142 |
|  | More than one race reported | -0.508 | 0.722 | -0.187 | 0.482 |
| Residence | Private residence | Reference |  |  |  |
|  | Nursing home | -1.611 | 1.009 | -0.318 | 0.110 |
|  | Homeless/homeless shelter | - | - |  | - |
|  | Other | 0.421 | 0.337 | 0.083 | 0.212 |
| Insurance | Private insurance | Reference |  |  |  |
|  | Medicare | -0.852 | 0.154 | -0.722 | <0.001 |
|  | Medicaid or CHIP or other state-based program | -0.808 | 0.114 | -0.685 | <0.001 |
|  | Worker’s compensation | 0.663 | 0.755 | 0.562 | 0.380 |
|  | Self-pay | -0.654 | 0.126 | -0.555 | <0.001 |
|  | No charge/Charity | -0.335 | 0.352 | -0.284 | 0.341 |
|  | Other | -0.981 | 0.328 | -0.832 | 0.003 |
| Visit year | 2005 | Reference |  |  |  |
|  | 2006 | -0.163 | 0.179 | -0.318 | 0.363 |
|  | 2007 | -0.201 | 0.179 | -0.392 | 0.260 |
|  | 2008 | -0.317 | 0.186 | -0.617 | 0.088 |
|  | 2009 | -0.558 | 0.188 | -1.087 | 0.003 |
|  | 2010 | -0.503 | 0.183 | -0.980 | 0.006 |
|  | 2011 | -0.300 | 0.178 | -0.584 | 0.093 |
|  | 2012 | -0.562 | 0.190 | -1.094 | 0.003 |
|  | 2013 | -1.049 | 0.232 | -2.045 | <0.001 |
|  | 2014 | -0.808 | 0.213 | -1.574 | <0.001 |
|  | 2015 | -0.968 | 0.235 | -1.885 | <0.001 |
|  | 2016 | 0.065 | 0.295 | 0.128 | 0.825 |
|  | 2017 | -0.943 | 0.253 | -1.838 | <0.001 |
| Visit month | January | Reference |  |  |  |
|  | February | -0.167 | 0.201 | -0.315 | 0.405 |
|  | March | -0.117 | 0.193 | -0.220 | 0.544 |
|  | April | -0.223 | 0.198 | -0.419 | 0.261 |
|  | May | -0.085 | 0.191 | -0.160 | 0.657 |
|  | June | -0.104 | 0.192 | -0.196 | 0.589 |
|  | July | -0.048 | 0.189 | -0.091 | 0.799 |
|  | August | -0.168 | 0.190 | -0.315 | 0.378 |
|  | September | -0.045 | 0.192 | -0.084 | 0.816 |
|  | October | -0.157 | 0.199 | -0.295 | 0.430 |
|  | November | -0.277 | 0.204 | -0.521 | 0.175 |
|  | December | -0.033 | 0.197 | -0.062 | 0.866 |
| Visit day | Sunday | Reference |  |  |  |
|  | Monday | -0.053 | 0.157 | -0.058 | 0.734 |
|  | Tuesday | 0.203 | 0.151 | 0.220 | 0.179 |
|  | Wednesday | 0.168 | 0.152 | 0.183 | 0.270 |
|  | Thursday | 0.081 | 0.156 | 0.089 | 0.603 |
|  | Friday | 0.274 | 0.153 | 0.299 | 0.073 |
|  | Saturday | 0.035 | 0.160 | 0.038 | 0.825 |
| Arrival time | Morning | Reference |  |  |  |
|  | Afternoon | -0.099 | 0.107 | -0.061 | 0.354 |
|  | Evening | -0.215 | 0.117 | -0.133 | 0.066 |
|  | Night | -0.212 | 0.113 | -0.131 | 0.061 |
| Temperature |  | 0.449 | 0.065 | 0.145 | <0.001 |
| Triage level | Immediate | Reference |  |  |  |
|  | Emergent | -0.542 | 0.262 | -0.194 | 0.038 |
|  | Urgent | -0.829 | 0.243 | -0.296 | 0.001 |
|  | Semi-urgent | -1.524 | 0.273 | -0.544 | <0.001 |
|  | Nonurgent | -1.571 | 0.375 | -0.561 | <0.001 |
| Is injury/poisoning intentional | Intentional | Reference |  |  |  |
|  | Unintentional | -1.244 | 0.796 | -0.202 | 0.118 |
|  | Questionable injury status | -1.533 | 0.895 | -0.249 | 0.087 |
| Visit related to an injury/poison/adverse effect of medical treatment with in 72 hours | No | Reference |  |  |  |
|  | Yes | -2.116 | 0.534 | -0.342 | <0.001 |
| Systolic BP |  | -0.001 | 0.002 | -0.016 | 0.608 |
| Diastolic BP |  | -0.011 | 0.003 | -0.107 | 0.002 |
| Pulse Oximetry |  | -0.005 | 0.002 | -0.054 | 0.022 |
| 72h Revisit | Yes | Reference |  |  |  |
|  | No | 0.317 | 0.222 | 0.038 | 0.154 |
| Pain level | Mild | Reference |  |  |  |
|  | Moderate | 0.669 | 0.148 | 0.285 | <0.001 |
|  | Very severe | 0.922 | 0.143 | 0.393 | <0.001 |
| Diagnostic services provided | No | Reference |  |  |  |
|  | Yes | 0.846 | 0.228 | 0.156 | <0.001 |

| **Table S4. Parameter estimation with structured variables of the logistic regression for pediatric ED patients, NHAMCS 2005-2017** | | | | | |
| --- | --- | --- | --- | --- | --- |
|  | | **Coefficient** | **Standard Error** | **Standardized Coefficient** | ***p* value** |
| Sex | Famale | Reference |  |  |  |
|  | Male | 1.225 | 0.145 | 0.338 | <0.001 |
| Age |  | 0.134 | 0.018 | 0.399 | <0.001 |
| Ethnicity | Hispanic or Latino | Reference |  |  |  |
|  | Not Hispanic or Latino | -0.249 | 0.154 | -0.063 | 0.105 |
| Race | White | Reference |  |  |  |
|  | Black/African American | -1.030 | 0.249 | -0.433 | <0.001 |
|  | Asian | -0.239 | 0.447 | -0.100 | 0.593 |
|  | Native Hawaiian/Other Pacific Islander | -0.109 | 0.761 | -0.046 | 0.886 |
|  | American Indian/Alaska Native | -0.659 | 1.037 | -0.277 | 0.525 |
|  | More than one race reported | 0.345 | 0.786 | 0.145 | 0.661 |
| Residence | Private residence | Reference |  |  |  |
|  | Nursing home | - | - | - | - |
|  | Homeless/homeless shelter | - | - | - | - |
|  | Other | 0.259 | 0.774 | 0.028 | 0.738 |
| Insurance | Private insurance | Reference |  |  |  |
|  | Medicare | -0.819 | 0.736 | -0.567 | 0.266 |
|  | Medicaid or CHIP or other state-based program | -0.295 | 0.150 | -0.204 | 0.049 |
|  | Worker’s compensation | - | - | - | - |
|  | Self-pay | -0.524 | 0.297 | -0.363 | 0.078 |
|  | No charge/Charity | - | - | - | - |
|  | Other | 0.177 | 0.433 | 0.123 | 0.683 |
| Visit year | 2005 | Reference |  |  |  |
|  | 2006 | -0.258 | 0.291 | -0.485 | 0.375 |
|  | 2007 | -0.657 | 0.327 | -1.235 | 0.044 |
|  | 2008 | -0.445 | 0.305 | -0.836 | 0.144 |
|  | 2009 | -0.695 | 0.293 | -1.306 | 0.018 |
|  | 2010 | -1.386 | 0.355 | -2.604 | <0.001 |
|  | 2011 | -0.601 | 0.304 | -1.130 | 0.048 |
|  | 2012 | -0.769 | 0.309 | -1.444 | 0.013 |
|  | 2013 | -0.639 | 0.322 | -1.200 | 0.047 |
|  | 2014 | -0.937 | 0.330 | -1.760 | 0.004 |
|  | 2015 | -0.935 | 0.344 | -1.756 | 0.007 |
|  | 2016 | -1.141 | 0.488 | -2.143 | 0.019 |
|  | 2017 | -0.985 | 0.360 | -1.851 | 0.006 |
| Visit month | January | Reference |  |  |  |
|  | February | -0.036 | 0.330 | -0.069 | 0.913 |
|  | March | 0.011 | 0.346 | 0.022 | 0.974 |
|  | April | -0.077 | 0.347 | -0.149 | 0.824 |
|  | May | 0.308 | 0.322 | 0.594 | 0.339 |
|  | June | 0.088 | 0.344 | 0.169 | 0.799 |
|  | July | 0.051 | 0.351 | 0.099 | 0.884 |
|  | August | 0.171 | 0.323 | 0.329 | 0.598 |
|  | September | 0.855 | 0.291 | 1.647 | 0.003 |
|  | October | 0.613 | 0.302 | 1.181 | 0.042 |
|  | November | 0.099 | 0.338 | 0.192 | 0.769 |
|  | December | 0.265 | 0.340 | 0.512 | 0.435 |
| Visit day | Sunday | Reference |  |  |  |
|  | Monday | -0.138 | 0.248 | -0.154 | 0.579 |
|  | Tuesday | -0.302 | 0.264 | -0.337 | 0.252 |
|  | Wednesday | 0.241 | 0.240 | 0.270 | 0.315 |
|  | Thursday | 0.087 | 0.246 | 0.097 | 0.725 |
|  | Friday | 0.005 | 0.256 | 0.006 | 0.984 |
|  | Saturday | 0.029 | 0.250 | 0.032 | 0.908 |
| Arrival time | Morning | Reference |  |  |  |
|  | Afternoon | 0.326 | 0.194 | 0.201 | 0.093 |
|  | Evening | -0.053 | 0.202 | -0.033 | 0.794 |
|  | Night | -0.048 | 0.197 | -0.029 | 0.808 |
| Temperature |  | 0.041 | 0.084 | 0.024 | 0.625 |
| Triage level | Immediate | Reference |  |  |  |
|  | Emergent | -0.240 | 0.480 | -0.095 | 0.617 |
|  | Urgent | -0.945 | 0.449 | -0.372 | 0.036 |
|  | Semi-urgent | -1.524 | 0.475 | -0.601 | 0.001 |
|  | Nonurgent | -1.386 | 0.575 | -0.546 | 0.016 |
| Is injury/poisoning intentional | Intentional | Reference |  |  |  |
|  | Unintentional | - | - | - | - |
|  | Questionable injury status | - | - | - | - |
| Visit related to an injury/poison/adverse effect of medical treatment with in 72 hours | No | Reference |  |  |  |
|  | Yes | - | - | - | - |
| Systolic BP |  | 0.005 | 0.005 | 0.040 | 0.328 |
| Diastolic BP |  | -0.017 | 0.006 | -0.478 | 0.003 |
| Pulse Oximetry |  | 0.006 | 0.003 | 0.105 | 0.074 |
| 72h Revisit | Yes | Reference |  |  |  |
|  | No | -0.120 | 0.347 | -0.013 | 0.730 |
| Pain level | Mild | Reference |  |  |  |
|  | Moderate | 1.324 | 0.207 | 0.547 | <0.001 |
|  | Very severe | 1.551 | 0.221 | 0.641 | <0.001 |
| Diagnostic services provided | No | Reference |  |  |  |
|  | Yes | 1.067 | 0.248 | 0.280 | <0.001 |


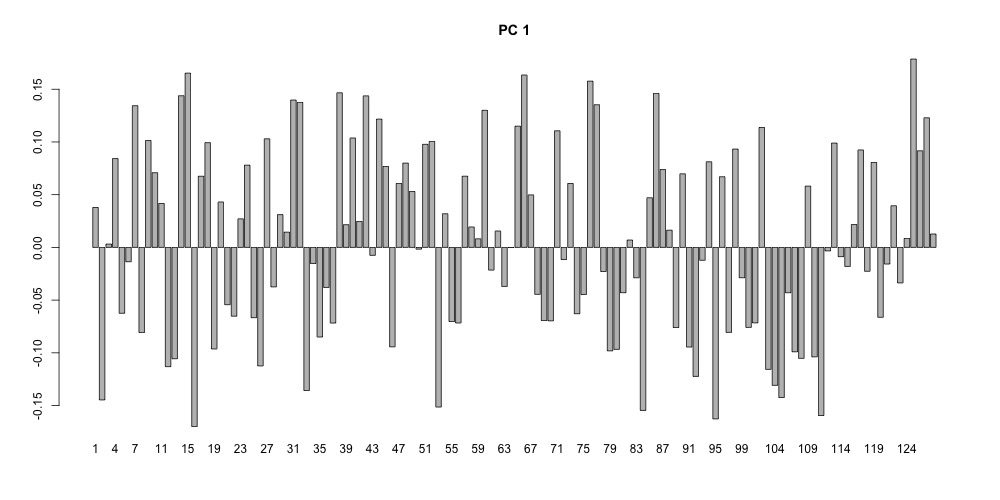

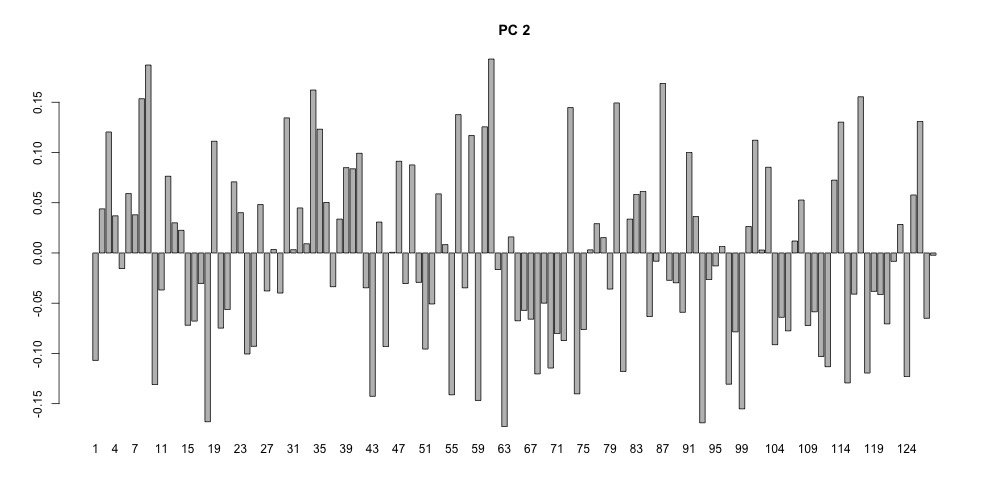

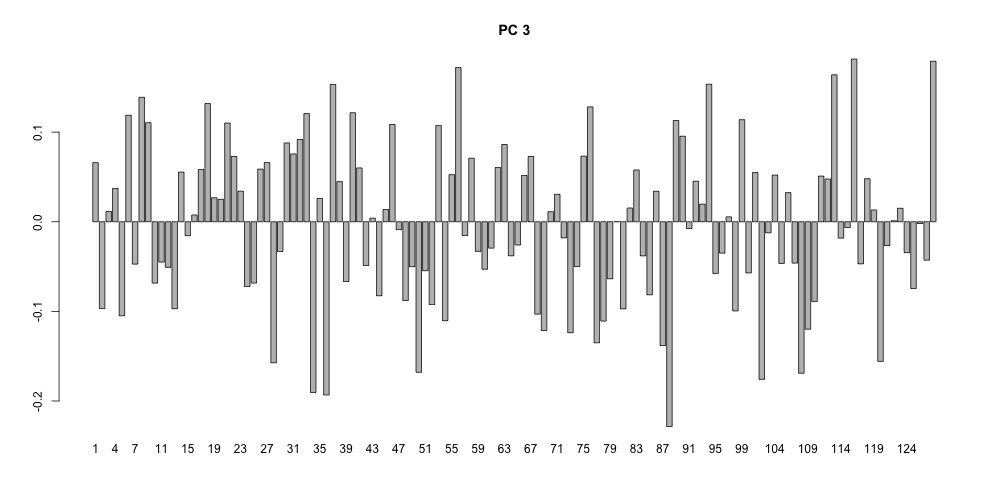

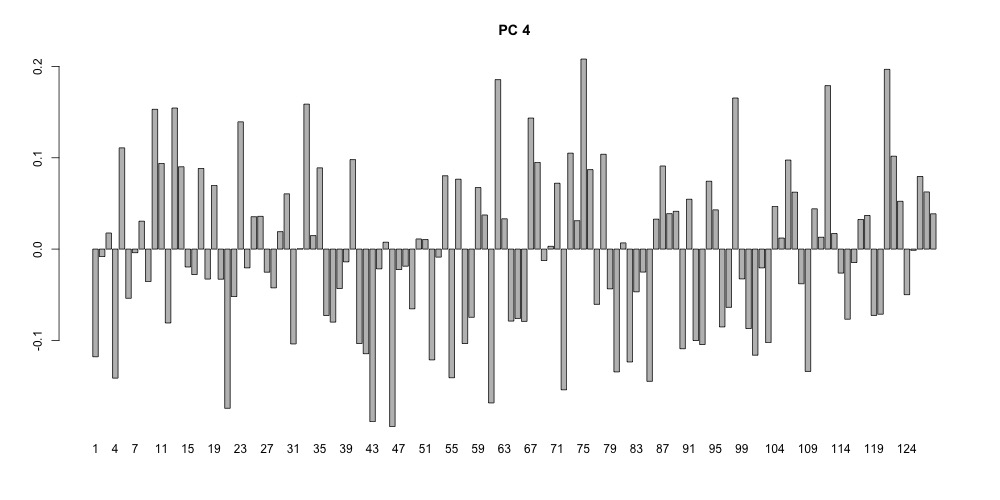

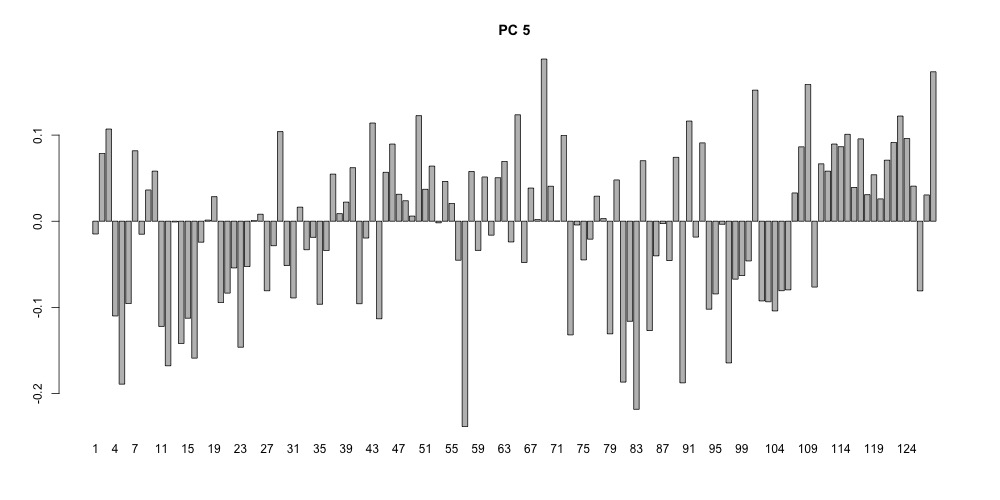

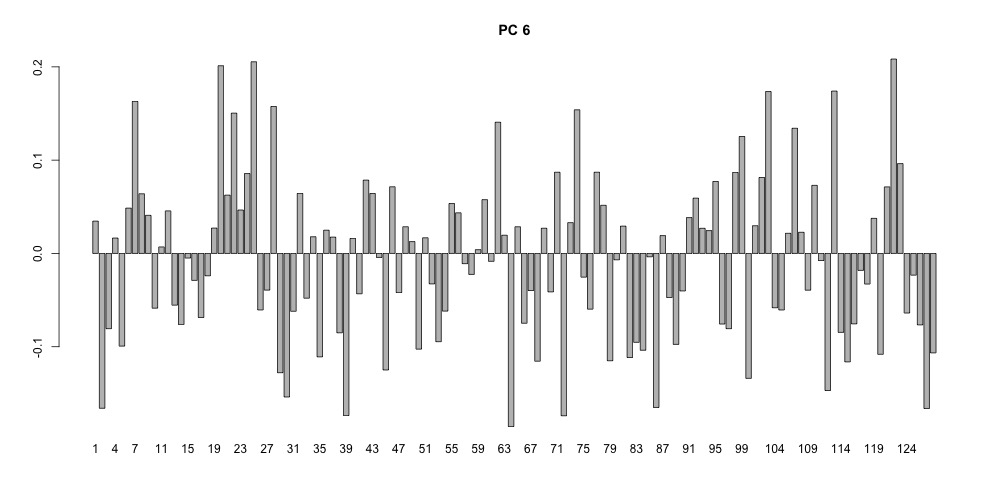

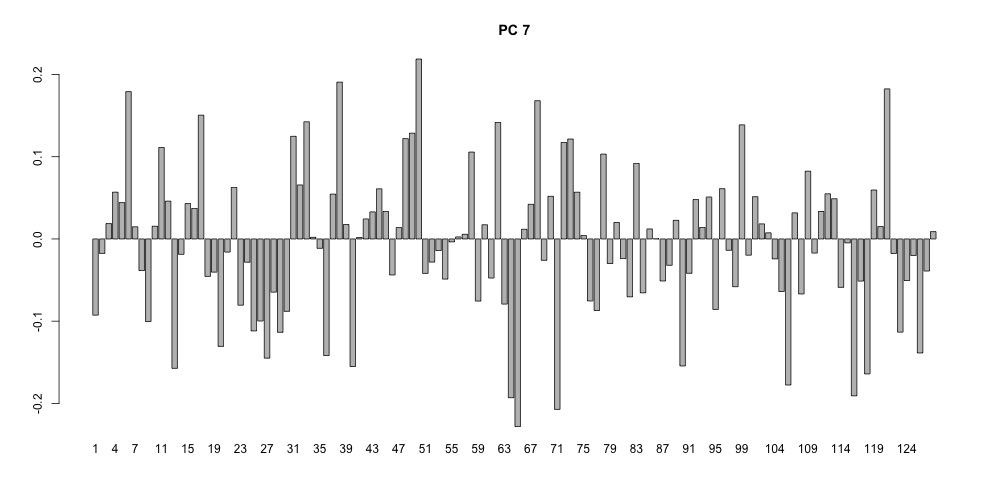

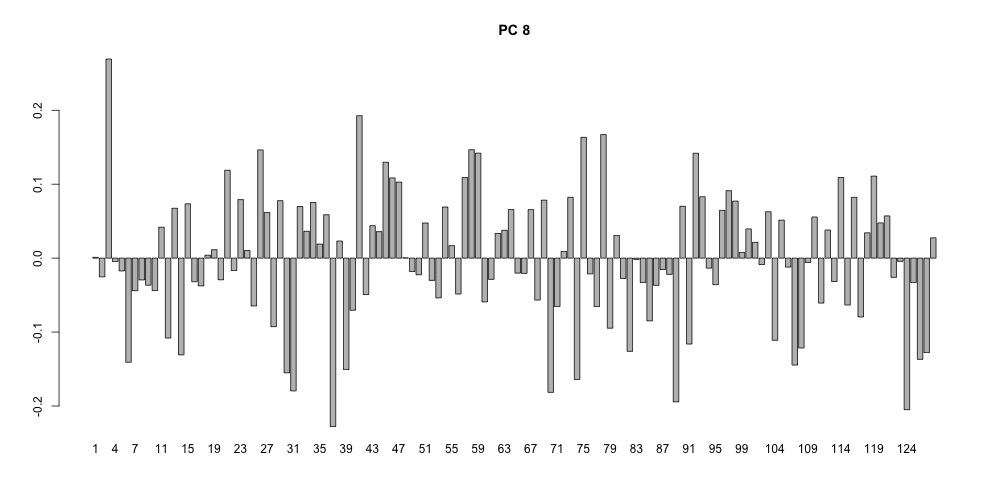

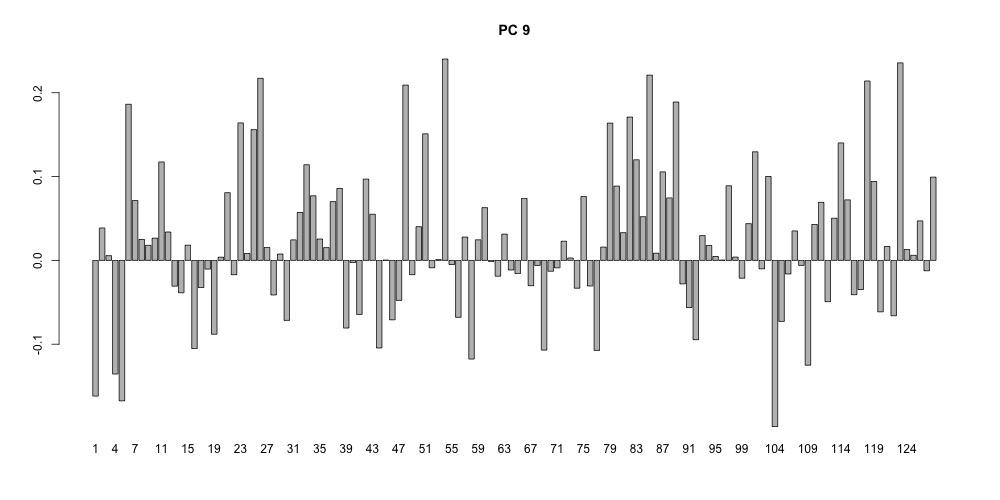

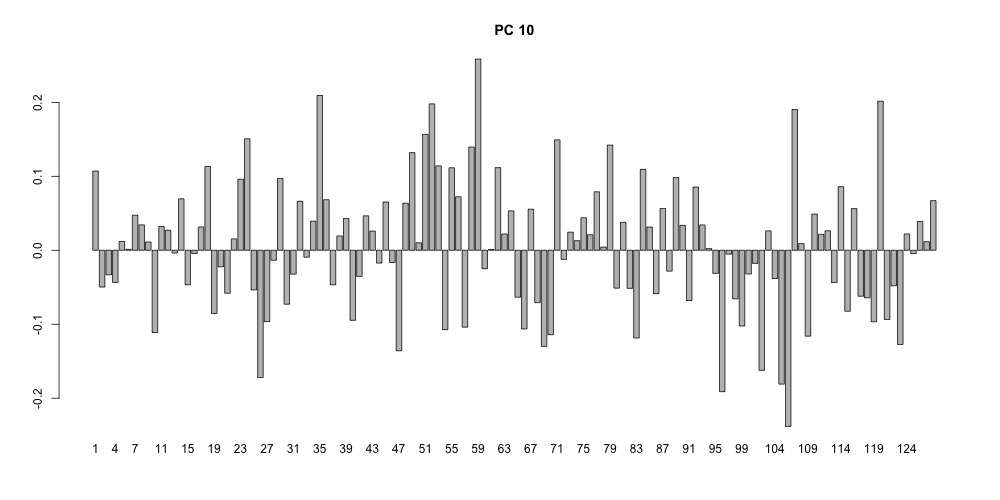

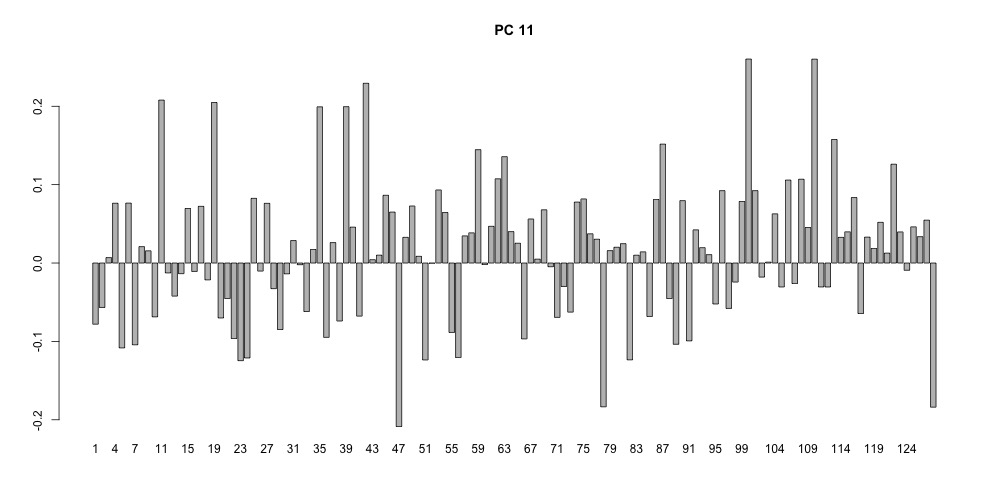

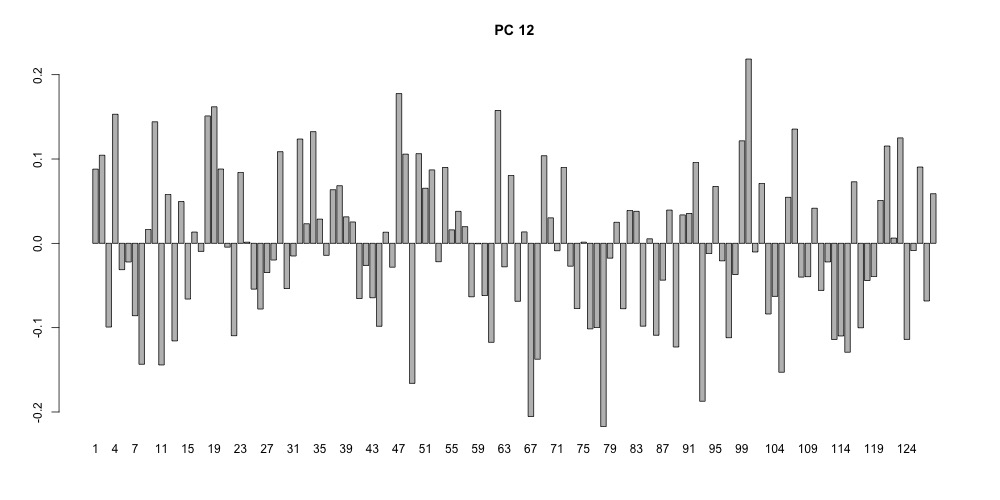

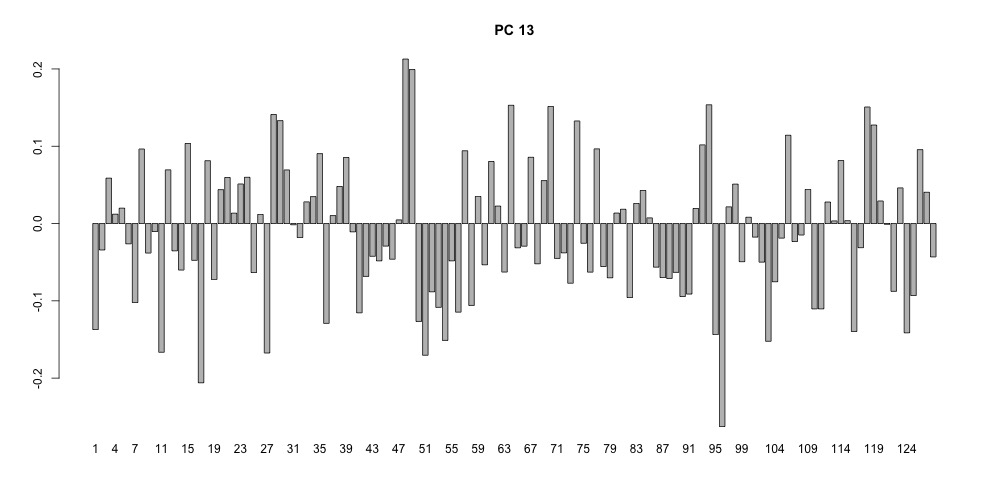

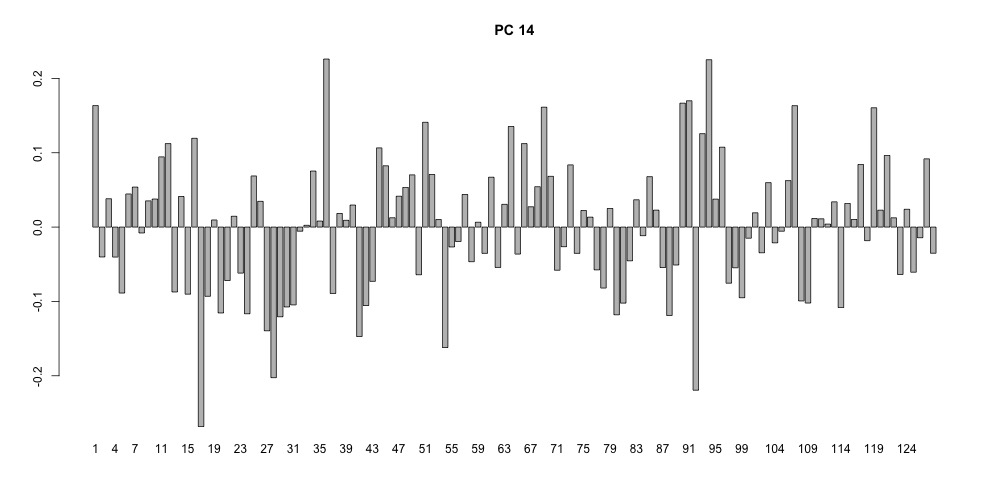

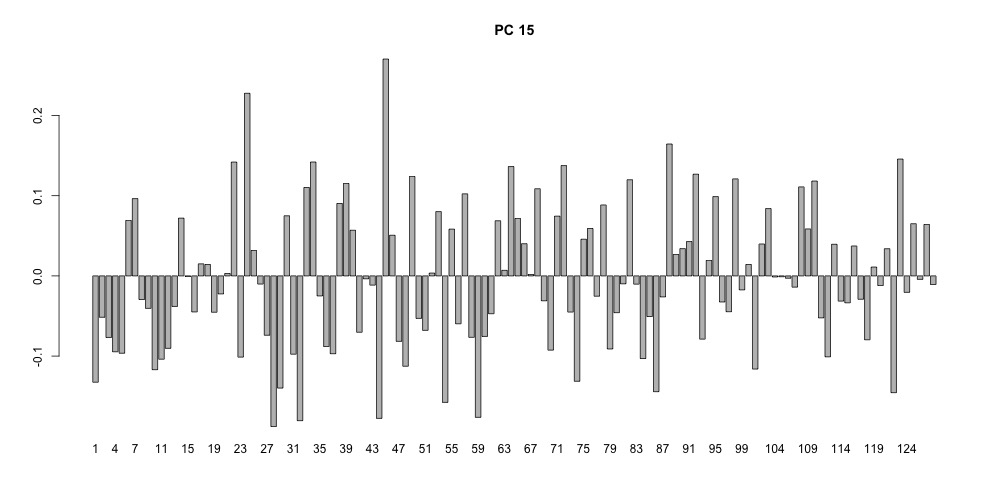

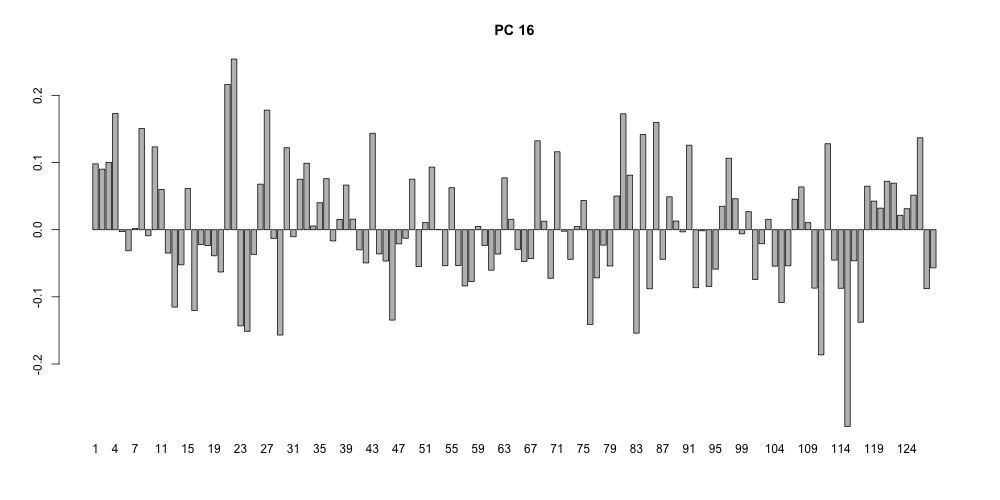

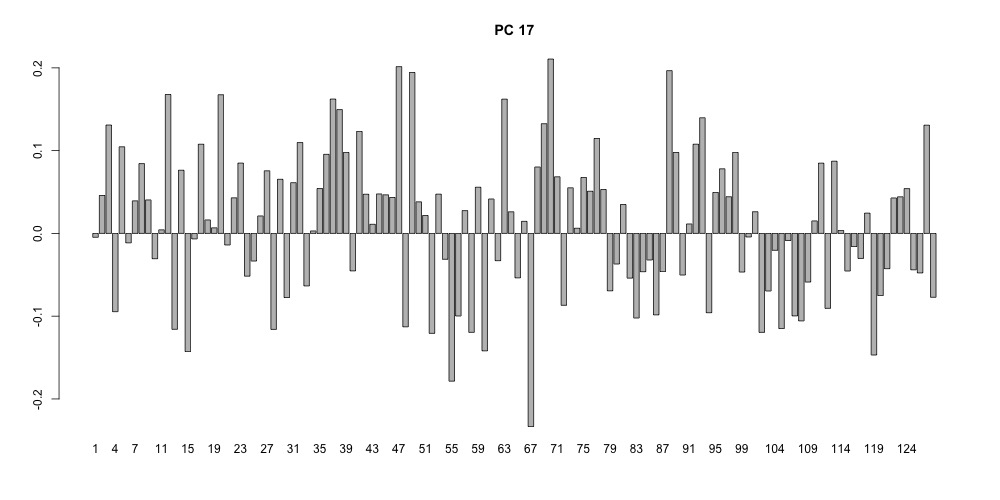

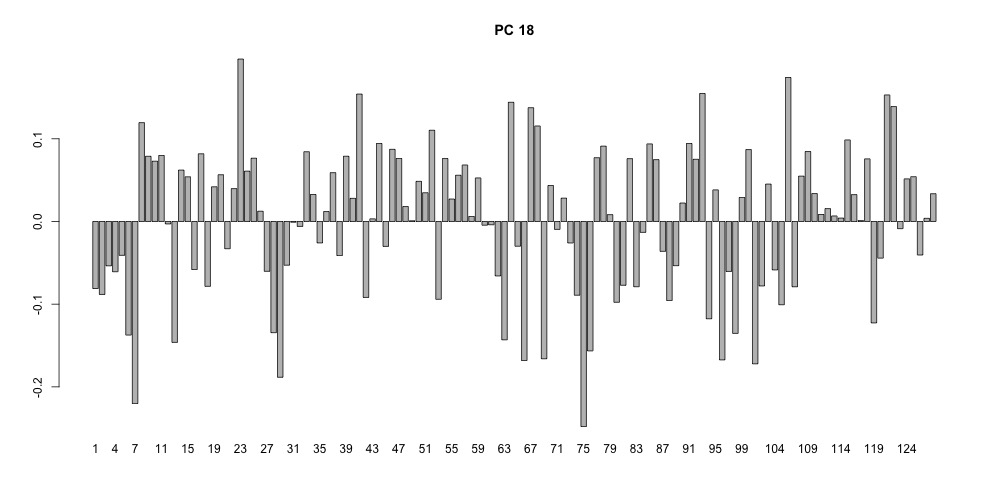

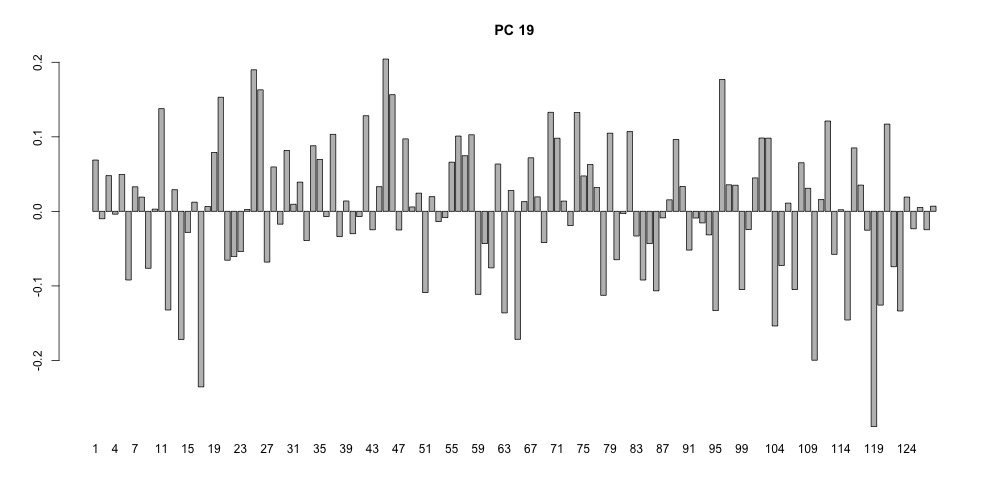

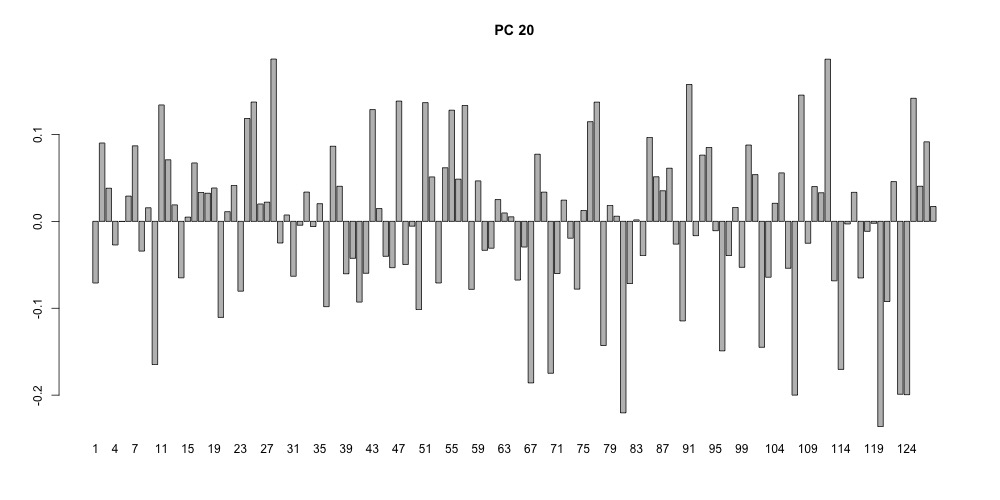

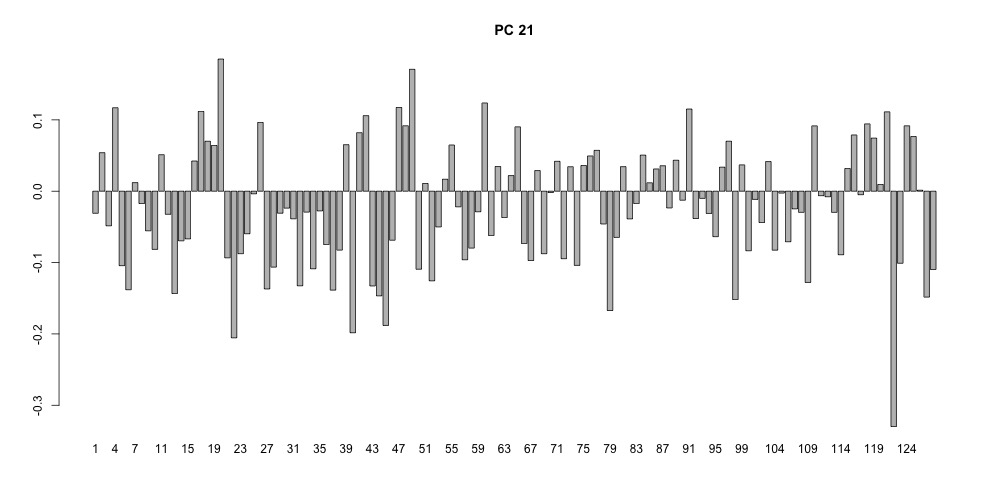

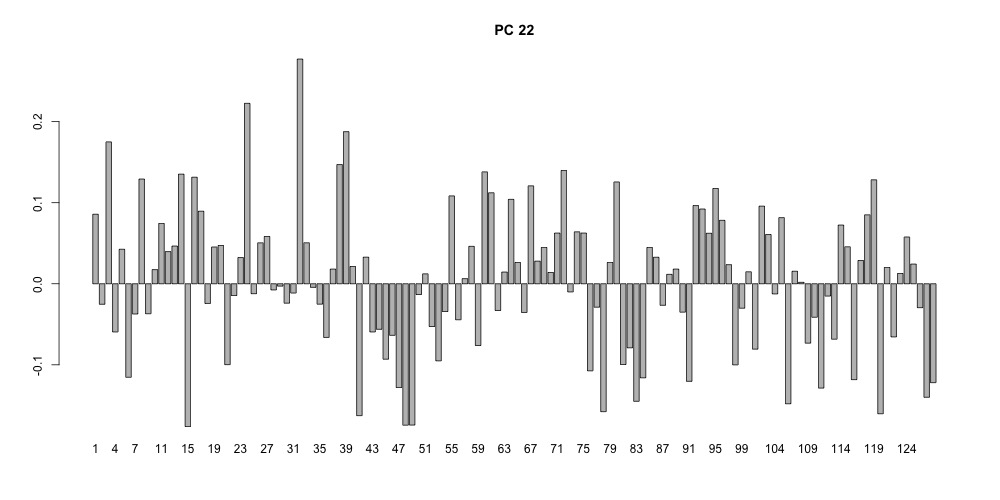

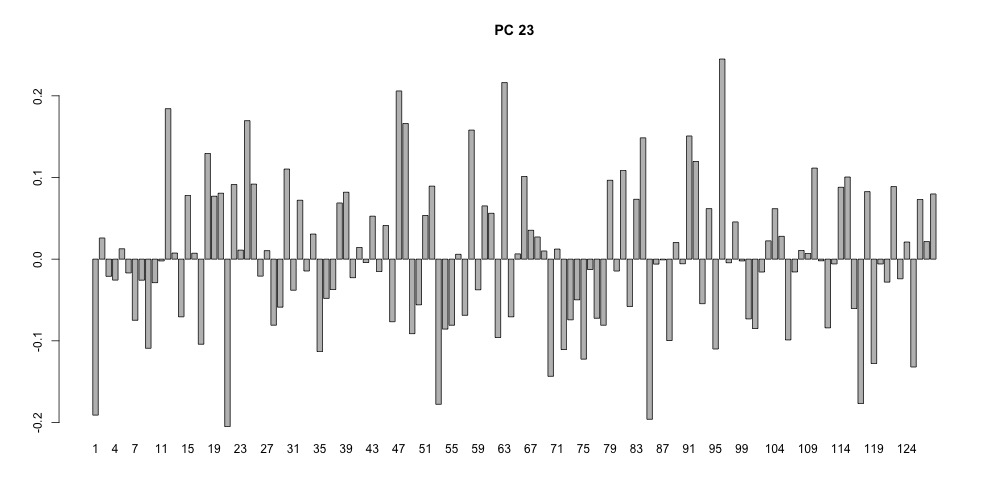

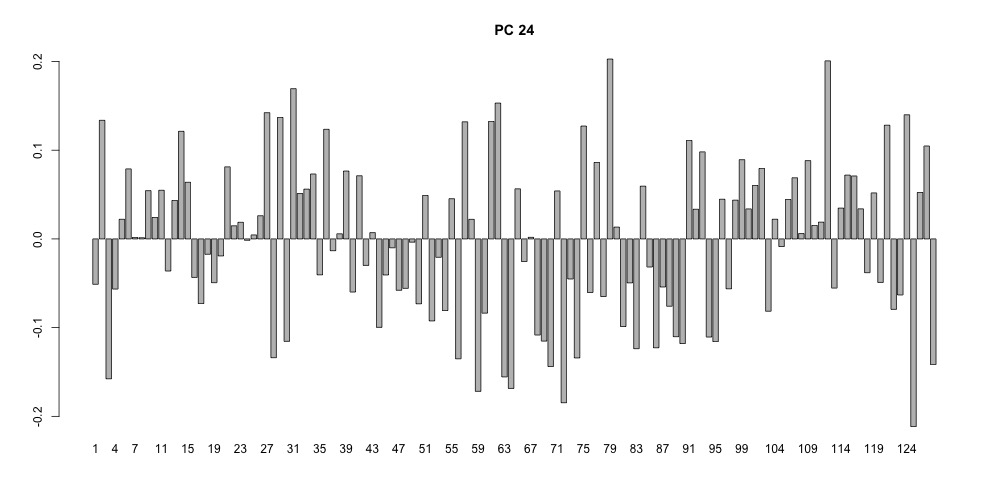


**Figure S1. The contribution (weights) of each 128 Doc2Vec output to the first 24 principle components**
